# A comprehensive investigation of mesophotic coral ecosystems in the Hawaiian Archipelago

**DOI:** 10.7717/peerj.2475

**Published:** 2016-10-04

**Authors:** Richard L. Pyle, Raymond Boland, Holly Bolick, Brian W. Bowen, Christina J. Bradley, Corinne Kane, Randall K. Kosaki, Ross Langston, Ken Longenecker, Anthony Montgomery, Frank A. Parrish, Brian N. Popp, John Rooney, Celia M. Smith, Daniel Wagner, Heather L. Spalding

**Affiliations:** 1 Natural Sciences, Bernice Pauahi Bishop Museum, Honolulu, HI, United States; 2 Pacific Islands Fisheries Science Center, National Oceanographic and Atmospheric Administration, Honolulu, HI, United States; 3 Hawai‘i Pacific University, Honolulu, HI, United States; 4 Hawai‘i Institute of Marine Biology, University of Hawai‘i at Manoa, Honolulu, HI, United States; 5 Life and Environmental Sciences, University of California at Merced, Merced, CA, United States; 6 Department of Oceanography, University of Hawai‘i at Manoa, Honolulu, HI, United States; 7 Environmental and Natural Resource Sciences, Washington State University, Pullman, WA, United States; 8 Papahānaumokuākea Marine National Monument, National Oceanic and Atmospheric Administration, Honolulu, HI, United States; 9 Pacific Islands Fish and Wildlife Office, U.S. Fish and Wildlife Service, Honolulu, HI, United States; 10 Department of Geology and Geophysics, University of Hawai‘i at Manoa, Honolulu, HI, United States; 11 Joint Institute for Marine and Atmospheric Research, University of Hawai‘i at Manoa, Honolulu, HI, United States; 12 Department of Botany, University of Hawai‘i at Manoa, Honolulu, HI, United States

**Keywords:** Mesophotic coral ecosystems, Hawaiian Archipelago, Endemism, Refugia, Closed-circuit rebreathers, Amino acid isotopic composition

## Abstract

Although the existence of coral-reef habitats at depths to 165 m in tropical regions has been known for decades, the richness, diversity, and ecological importance of mesophotic coral ecosystems (MCEs) has only recently become widely acknowledged. During an interdisciplinary effort spanning more than two decades, we characterized the most expansive MCEs ever recorded, with vast macroalgal communities and areas of 100% coral cover between depths of 50–90 m extending for tens of km^2^ in the Hawaiian Archipelago. We used a variety of sensors and techniques to establish geophysical characteristics. Biodiversity patterns were established from visual and video observations and collected specimens obtained from submersible, remotely operated vehicles and mixed-gas SCUBA and rebreather dives. Population dynamics based on age, growth and fecundity estimates of selected fish species were obtained from laser-videogrammetry, specimens, and otolith preparations. Trophic dynamics were determined using carbon and nitrogen stable isotopic analyses on more than 750 reef fishes. MCEs are associated with clear water and suitable substrate. In comparison to shallow reefs in the Hawaiian Archipelago, inhabitants of MCEs have lower total diversity, harbor new and unique species, and have higher rates of endemism in fishes. Fish species present in shallow and mesophotic depths have similar population and trophic (except benthic invertivores) structures and high genetic connectivity with lower fecundity at mesophotic depths. MCEs in Hawai‘i are widespread but associated with specific geophysical characteristics. High genetic, ecological and trophic connectivity establish the potential for MCEs to serve as refugia for some species, but our results question the premise that MCEs are more resilient than shallow reefs. We found that endemism within MCEs increases with depth, and our results do not support suggestions of a global faunal break at 60 m. Our findings enhance the scientific foundations for conservation and management of MCEs, and provide a template for future interdisciplinary research on MCEs worldwide.

## Introduction

Tropical coral reefs are compelling subjects for a wide range of scientific investigations because they provide an optimal combination of high diversity, extensive existing data, robust information infrastructure, large potential for the discovery of new taxa, and opportunities to gain new insights into fundamental ecological dynamics (Reaka-Kudla, 1997). They are also among the most severely threatened ecosystems on Earth (Pandolfi et al., 2003; Knowlton et al., 2010). It has become increasingly evident in recent years that anthropogenic impacts, such as overharvesting, pollution, coastal development, invasive species, ocean acidification, and global climate change, imperil the health of coral-reef ecosystems worldwide (Bruno & Selig, 2007; Burke et al., 2011). Although the vast majority of known hermatypic coral reefs occur at depths of less than 40 m, there is longstanding evidence for photosynthetic corals and associated reef communities at greater depths. Zooxanthellate hermatypic corals have been found at 98 m in the tropical Atlantic (Hartman, 1973; Fricke & Meischne, 1985; Reed, 1985), below 100 m in the Caribbean (Locker et al., 2010; Bongaerts et al., 2015; Garcia-Sais et al., 2014; Smith et al., 2014), 112 m at Enewetak (Colin et al., 1986), 125 m on the Great Barrier Reef (Englebert et al., 2014), 145 m in the Red Sea (Fricke & Schuhmacher, 1983), 153 m in Hawai‘i, and 165 m at Johnston Atoll (Strasburg, Jones & Iversen, 1968; Maragos & Jokiel, 1985; Kahng & Maragos, 2006). Hopley (1991) reported 100% coral cover at 70 m on the Great Barrier Reef, and Jarrett et al. (2005) reported up to 60% coral cover at 60–75 m at Pulley Ridge in the Gulf of Mexico. Photosynthetic algae have been observed at similar or deeper depths (Porter, 1973; Littler et al., 1985; Colin et al., 1986; Hills-Colinvaux, 1986), and fish species at such depths belong almost exclusively to families typical of shallower coral-reef environments (Pyle, 1996b; Pyle, 1999a). Despite these scattered reports, coral-reef environments at depths greater than 30 m are poorly characterized, largely because of the logistical difficulties associated with accessing such depths (Pyle, 1996c; Pyle, 1998; Pyle, 1999b; Pyle, 2000; Parrish & Pyle, 2001). There are potentially thousands of species that have yet to be discovered and scientifically described from deeper coral reef habitats (Pyle, 1996d; Pyle, 2000; Rowley, 2014) and the basic ecology and population dynamics of these communities, as well as their connectivity with shallow reefs, are just beginning to be explored.

Most coral-reef monitoring programs are designed to target shallow reefs (Jokiel et al., 2001; Brown et al., 2004; Preskitt, Vroom & Smith, 2004; Kenyon et al., 2006). In recent years, there has been a greater effort to document coral-reef ecosystems at depths of 30 to over 150 m, now referred to as “Mesophotic Coral Ecosystems” (MCEs) (Hinderstein et al., 2010; Baker, Puglise & Harris, 2016). These research efforts have primarily focused on aspects of MCEs that are relevant to management policies, such as their distribution, ecology and biodiversity, as MCEs have been identified as a conservation priority (Blyth-Skyrme et al., 2013; Sadovy de Mitcheson et al., 2013). However, despite the growing body of research targeting MCEs, they are often not included in reef assessment and monitoring programs, management-related reports on the status and health of coral reefs (Brainard et al., 2003), or general overviews of coral-reef science (Trenhaile, 1997). Most studies of coral-reef development (and the models derived from them) (Dollar, 1982; Grigg, 1998; Braithwaite et al., 2000; Rooney et al., 2004) and coral-reef ecology (Luckhurst & Luckhurst, 1978; Friedlander & Parrish, 1998; Friedlander & DeMartini, 2002; Friedlander et al., 2003) do not include MCEs. Indeed, most of our understanding of coral-reef ecosystems is biased by the preponderance of data from depths less than 30 m, which represents less than one-fifth of the total depth range of the tropical coral-reef environment (Pyle, 1996b; Pyle, 1999a). An understanding of MCEs is essential to successfully characterize the health of coral reefs in general, and to formulate effective management plans in the face of increasing anthropogenic stress.

**Figure 1:**
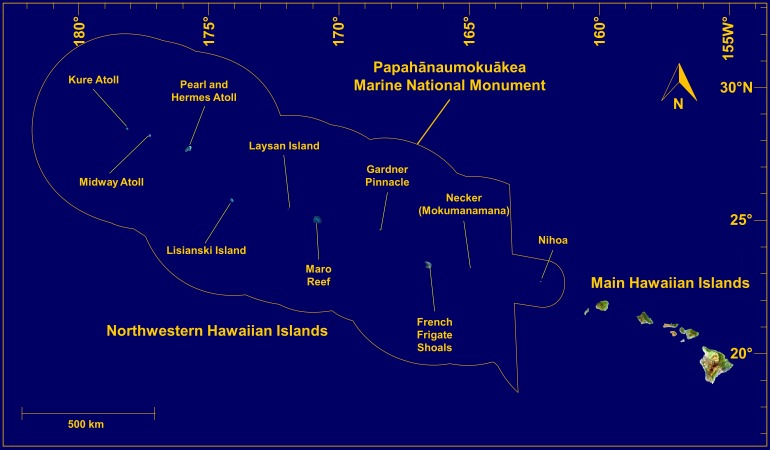
Map of the Hawaiian Archipelago. Source Imagery: Landsat.

Coral-reef environments within the Hawaiian Archipelago have been extensively studied and documented for decades (Maragos, 1977; Chave & Malahoff, 1998; Hoover, 1998; Mundy, 2005; Randall, 2007; Fletcher et al., 2008; Grigg et al., 2008; Jokiel, 2008; Rooney et al., 2008; Toonen et al., 2011; Selkoe et al., 2016). These islands and reefs stretch over 2,500 km across the north-central tropical Pacific Ocean, and consist of the eight Main Hawaiian Islands (MHI) in the southeast, and a linear array of uninhabited rocky islets, atolls, reefs, and seamounts comprising the Northwestern Hawaiian Islands (NWHI) (Fig. 1). Many Hawaiian reefs are protected by local, state and federal laws, with a wide range of management and conservation efforts already in place. In particular, the NWHI fall within the Papahānaumokuākea Marine National Monument, a federally protected area larger than all U.S. National Parks combined (>360,000 km^2^), which is listed as a World Heritage site and includes about 10% of coral-reef habitats within U.S. territorial waters (Rohmann et al., 2005).

The first investigations of MCEs within the Hawaiian Archipelago were conducted in the 1960s with SCUBA (Grigg, 1965) and submersibles (Brock & Chamberlain, 1968; Strasburg, Jones & Iversen, 1968). These early investigations found an unexpected abundance of reef-associated species (including hermatypic corals) at depths from 25 to 180 m. These studies also revealed that many species of fishes previously believed to be restricted to shallow water inhabit much greater depths than expected. In the decades that followed, a smattering of publications reported on MCEs within the Hawaiian Archipelago (Grigg, 1976; Agegian & Abbott, 1985; Maragos & Jokiel, 1985; Moffitt, Parrish & Polovina, 1989; Chave & Mundy, 1994; Parrish & Polovina, 1994; Pyle & Chave, 1994), but most of these involved either a few individual species or habitats, or focused on a broader depth range (with MCEs representing only a small portion of the study). Beginning in the late 1980s, the advent of “technical” mixed-gas diving opened up new opportunities for exploration of MCEs in Hawai‘i and elsewhere (Pyle, 1996a; Pyle, 1999b; Pyle, 2000; Grigg et al., 2002; Parrish & Pyle, 2002; Pence & Pyle, 2002; Parrish & Boland, 2004; Boland & Parrish, 2005; Grigg, 2006). In 2006, the discovery of extensive MCEs with near-100% coral cover off Maui, coupled with interest in documenting MCEs in the NWHI and a growing infrastructure supporting mixed-gas diving operations among Hawaiian research institutions, led to a surge of research in these deep-reef environments and a series of collaborative, multi-disciplinary projects dedicated to improving the understanding of MCEs. These projects include (1) the Deep Coral Reef Ecosystem Studies (Deep-CRES) program focused on the MCEs of the ‘Au‘au Channel off Maui and their relationship to shallower reefs funded by the National Oceanic and Atmospheric Administration’s (NOAA) Center for Sponsored Coastal Ocean Research, (2) two separate studies funded by NOAA’s Coral Reef Conservation Program to study MCEs off Kaua‘i and O‘ahu, and (3) ongoing annual research cruises sponsored by NOAA’s Office of National Marine Sanctuaries to study MCEs within the Papahānaumokuākea Marine National Monument. These projects, as well as many other smaller surveys over the past two decades, have provided an opportunity for a coordinated effort to explore and document MCEs across the Hawaiian Archipelago.

The overarching goal of these activities was to establish a baseline understanding of MCEs in Hawai‘i at depths ranging from 30 to over 150 m, and to provide insights into the structure, composition, ecological dynamics, and management needs of MCEs in general. The primary research activities were driven by a series of hypotheses designed to reveal fundamental characteristics of Hawaiian MCEs and how they compare with both shallow reef habitats and non-MCE habitats at comparable depths. The questions behind these hypotheses involved characterizations in four general categories: (1) basic geophysical habitat (water clarity, temperature, photosynthetically active radiation [PAR], water movement, nutrient levels, and substrate type), (2) patterns of biodiversity (species composition, relative abundance, and overlap, as well as patterns of endemism), (3) population structure and dynamics (growth rates, impact from anthropogenic and natural disturbance, connectivity, disease levels, age structure, fecundity and production), and (4) broad ecological patterns (trophic dynamics and genetic connectivity). In addition, data from these studies were used extensively to develop a spatial model based on physical parameters and other factors to predict the occurrence of MCEs in Hawai‘i and globally (Costa et al., 2015). Ultimately, our hope is that the insights gained from this research, such as the predicted distribution and abundance of MCEs, the richness and uniqueness of the biodiversity they harbor, and the potential for MCEs to serve as refugia for overexploited biological resources on shallow reefs, will help guide future policy decisions in the conservation and management of marine resources in Hawai‘i and elsewhere.

## Materials and Methods

As this synthesis represents a broad summary of MCEs in Hawai‘i, based on the results of a multi-year interdisciplinary collaborative effort by many individuals, the methods involved are extensive and diverse. The following represents a summary of methods used throughout this study, particularly as they pertain to data not previously published elsewhere. More detailed descriptions of methods used during this study, including aspects that have been previously published, are included in Supplemental Information 1, and within cited publications. The State of Hawai‘i Department of Land and Natural Resources developed Special Activity Permits for the University of Hawai‘i and National Marine Fisheries Service for work related to this project that occurred within State of Hawai‘i waters. All sampling procedures and experimental manipulations were reviewed as part of obtaining the field permit. All vertebrates (fishes) were collected in accordance with University of Hawai‘i IACUC protocol 09-753-5, “Phylogeography and Evolution of Reef Fishes” (PI: Dr. Brian Bowen), including collection and euthanization by spear.

### Study sites

**Figure 2:**
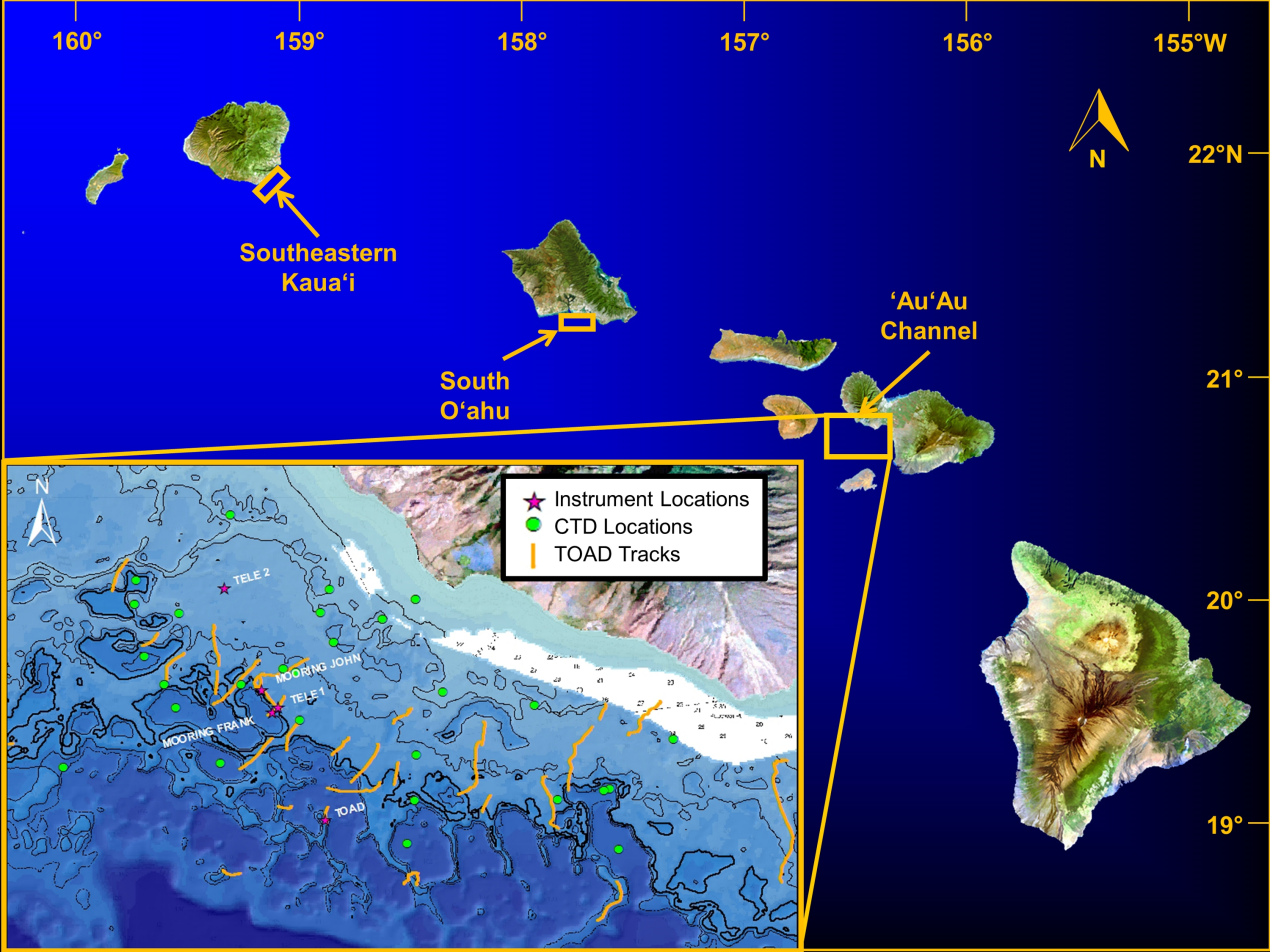
Location of study areas. Inset shows remote camera survey (TOAD) track locations, and sites for “John,” “Frank,” and “Tele 1” and “Tele 2” data moorings. MHI imagery from Landsat, USGS.

We examined MCEs at multiple sites throughout the Hawaiian Archipelago. The primary MHI study sites were in the ‘Au‘au Channel off Maui, southeast Kaua‘i, and the southern shore of O‘ahu (Fig. 2). Additional qualitative observations of MCEs around the islands of O‘ahu, Kaua‘i, Maui, and Hawai‘i provide complementary insights into general characteristics of MCEs in the MHI. Surveys of MCEs in the NWHI included visits to ten islands and reefs labelled in Fig. 1.

### Survey effort

**Figure 3:**
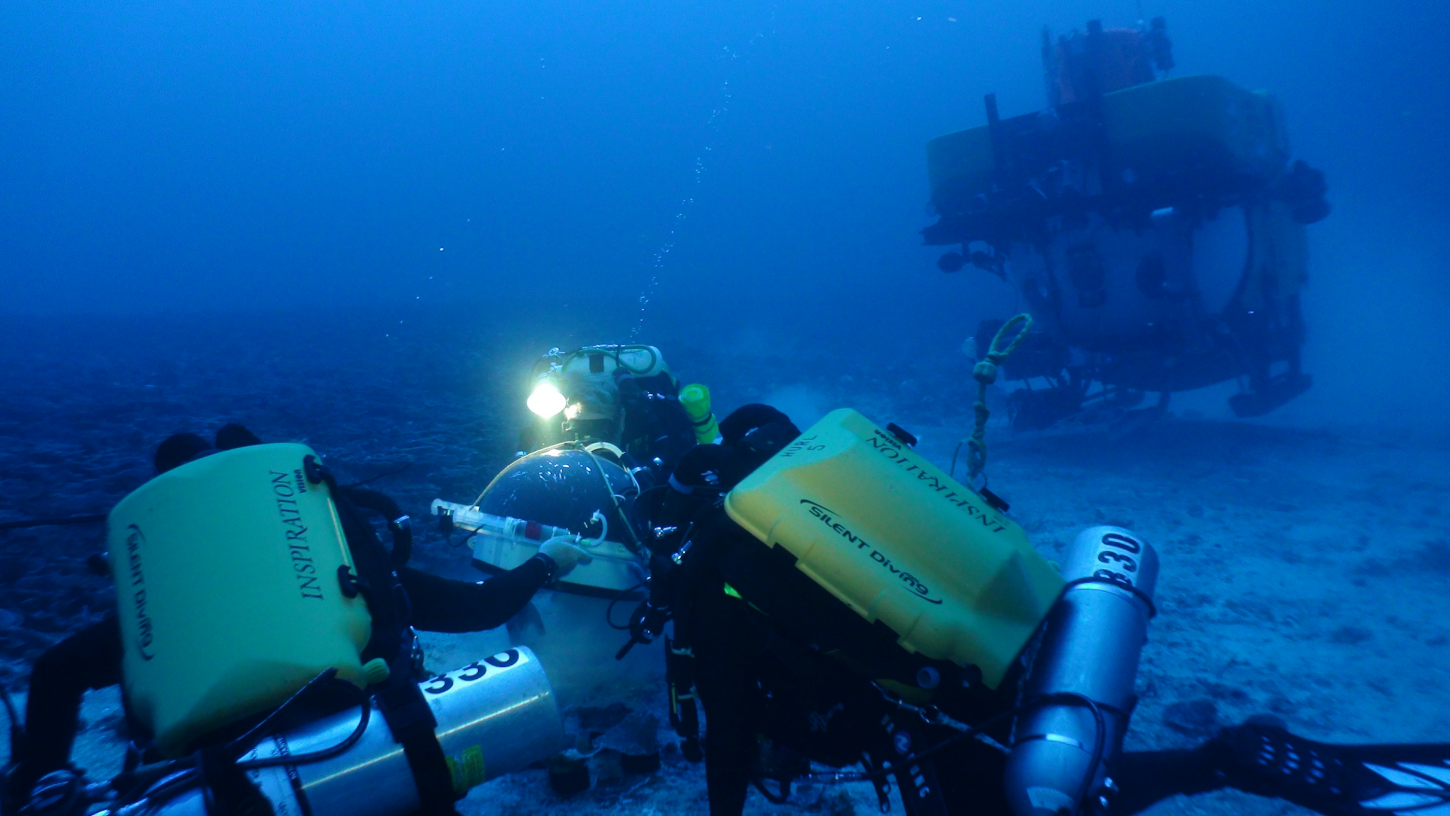
Research divers place a dome over a set of corals 89 m deep. Research divers Ken Longenecker (left), Dave Pence (center) and Christina Bradley (right) place a dome over a set of corals 89 m deep as part of an experiment to determine coral feeding patterns, while pilot Terry Kerby and science observers Brian Popp and Andrea Grottoli watch on from the HURL submersible *Pisces V*. Photo: RL Pyle.

Data from MCEs were gathered from mixed-gas rebreather dives, submersible dives, Remotely Operated Vehicle (ROV) dives, and Towed Optical Assessment Device (TOAD camera sled) transects. The submersible and ROV dives were conducted using the Hawai‘i Undersea Research Laboratory’s (HURL) *Pisces IV* and *Pisces V* submersibles, and the *RCV-150* ROV. On several occasions, both submersibles and rebreather divers conducted simultaneous, coordinated field operations (Fig. 3). Mixed-gas dives in the NWHI were conducted from the NOAA Ship *Hi‘ialakai*. Dive sites in the MHI were determined by a variety of factors, including previously known MCE habitat, bathymetry data, and direct site identification by submersible, ROV and TOAD; whereas dive sites in the NWHI targeted steep vertical drop-offs at depths of 50–85 m located using historical charts and new multibeam sonar data collected with the *Hi‘ialakai*.

**Table 1:** List of all temperature sensors deployed across Maui and Kaua‘i.

Location	Latitude	Longitude	Depth (m)	Deployment
Makaheuna Point, Kaua‘i	21°51.388N	159°26.003W	46	13 June 2009 to 12 July 2010
Makaheuna Point, Kaua‘i	21°51.388N	159°26.003W	63	13 June 2009 to 12 July 2010
Kipu Kai, Kaua‘i	21°52.460N	159°23.028W	57	17 June 2009 to 13 July 2010
Keyhole Pinnacle, Maui	20°56.437N	156°45.619W	70	6 April 2009 to 17 January 2010
Keyhole Pinnacle, Maui	20°56.452N	156°45.652W	88	6 April 2009 to 17 January 2010
Keyhole Pinnacle, Maui	20°56.452N	156°45.652W	102	6 April 2009 to 17 January 2010
Keyhole Pinnacle, Maui	20°56.454N	156°45.661W	116	6 April 2009 to 17 January 2010
Keyhole Pinnacle, Maui	20°56.478N	156°45.666W	134	6 April 2009 to 17 January 2010
Keyhole Pinnacle, Maui	20°56.478N	156°45.666W	160	6 April 2009 to 17 January 2010
Branching Coral Reef, Maui	20°49.300N	156°40.377W	58	14 December 2009 to 14 December 2010
Stone Walls, Maui	20°52.890N	156°43.794W	34	17 December 2009 to 13 December 2010
Stone Walls, Maui	20°52.890N	156°43.794W	34	17 December 2009 to 13 December 2010
Stone Walls, Maui	20°52.890N	156°43.794W	42	17 December 2009 to 13 December 2010
Stone Walls, Maui	20°52.890N	156°43.794W	46	17 December 2009 to 13 December 2010
Stone Walls, Maui	20°52.890N	156°43.794W	49	17 December 2009 to 13 December 2010
Stone Walls, Maui	20°52.890N	156°43.794W	58	17 December 2009 to 13 December 2010

### Geophysical habitat characterization

The majority of geophysical habitat characterization focused on the ‘Au‘au Channel site (Fig. 2, inset). Existing moderate-resolution (20-m grid) bathymetry was supplemented with multibeam surveys at a resolution of 5 m in a few areas. Video transects from submersible, ROV and TOAD dives were used to document the spatial distribution of corals and macroalgal communities across >140 linear km of habitat (Fig. 2). Two specific areas were selected for detailed physical oceanographic characterization using four oceanographic moorings with temperature and pressure loggers, current meter, and current profiler. Additional temperature data were recorded at four sites between Maui and Kaua‘i (Table 1). Underwater irradiance was measured by lowering a calibrated spherical (4*π*) quantum sensor (Underwater LI-193SA, LI-COR, Lincoln, NE, USA) through the water via a profiling rig (*n* = 6 profiles taken within one hour of noon on 4, 5, and 6 August 2008 and 12, 13, and 14 July 2010 over dense *Leptoseris* spp. reefs at a maximum depth of 91 m off west Maui); data were stored with a LI-COR LI-1400 datalogger.

### Biodiversity patterns

**Figure 4:**
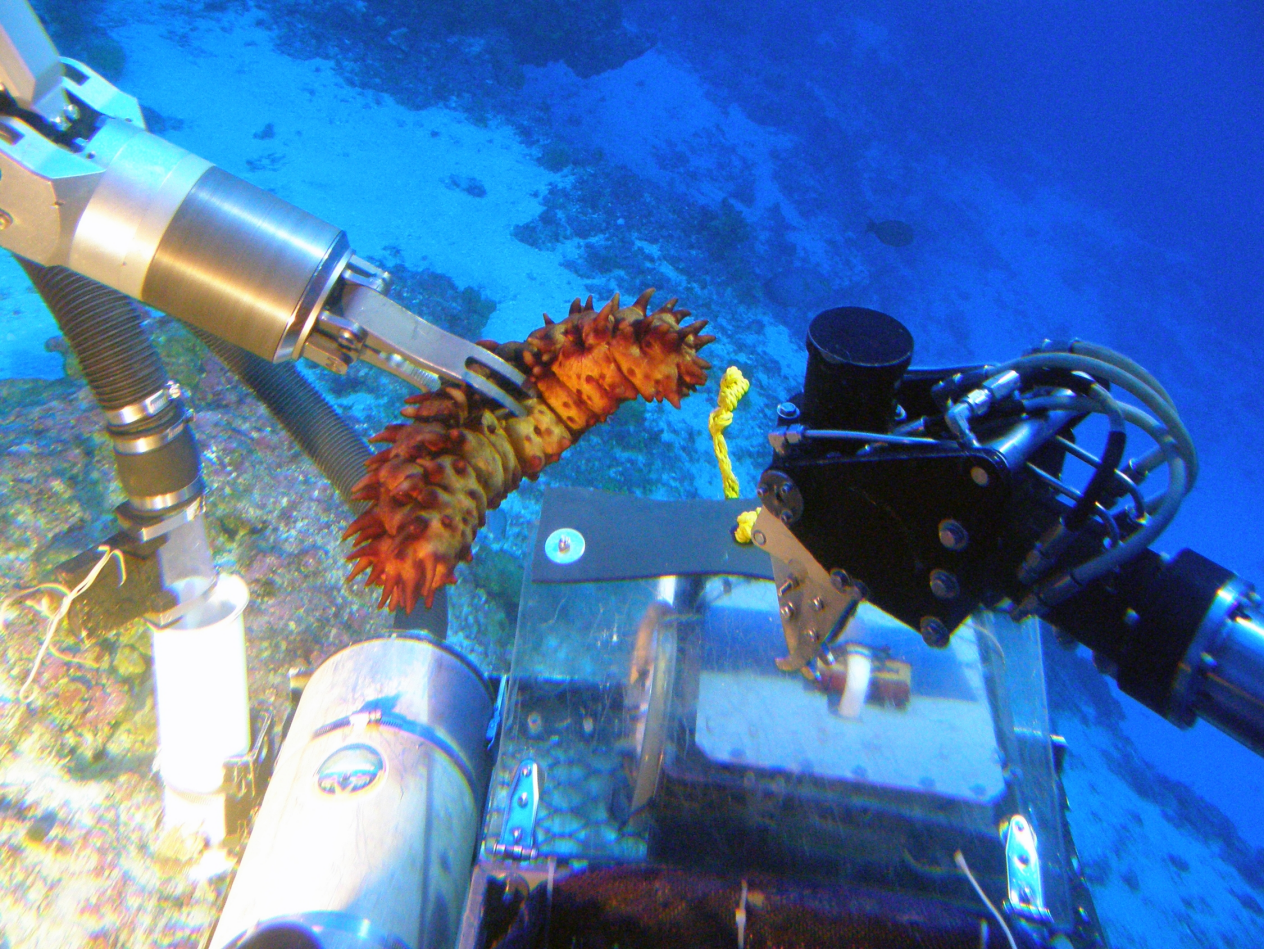
Collecting samples using the *Pisces* submersible manipulator arm. Photo: HURL.

The biodiversity surveys focused primarily on macroalgae, fishes, marine invertebrates, and corals. Surveys were conducted using a variety of visual, video, and collecting techniques. Direct visual observations were made by trained individuals during mixed-gas SCUBA dives and from submersibles. Videotapes were generated by divers, submersibles, ROV, and TOAD camera system. Specimens were collected by divers and the manipulator arm of the *Pisces* submersibles (Fig. 4). Qualitative collections and observations were made to determine species presence, and quantitative transects were made to measure the distribution and abundance of species. Depth ranges for algae and fishes known to occur at depths of less than 200 m were determined from quantitative and qualitative surveys, and available published information. All specimens were photo-documented using high-resolution digital cameras, and voucher specimens were deposited in the Bishop Museum Natural Sciences collections.

**Figure 5:**
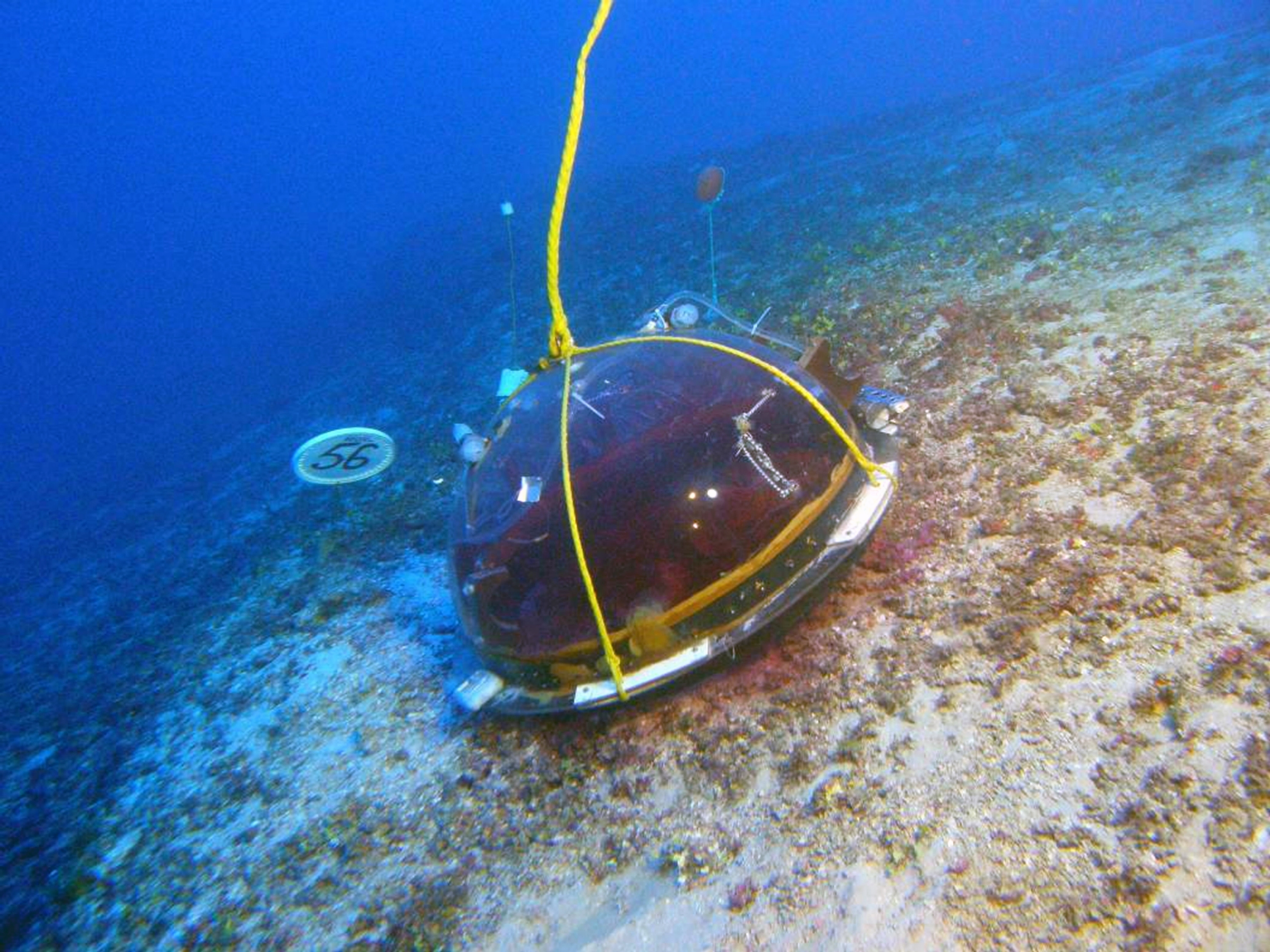
Colony of *Leptoseris* sp. being stained with Alizarin Red for growth rate studies. Photo: HURL.

### Population dynamics

**Figure 6:**
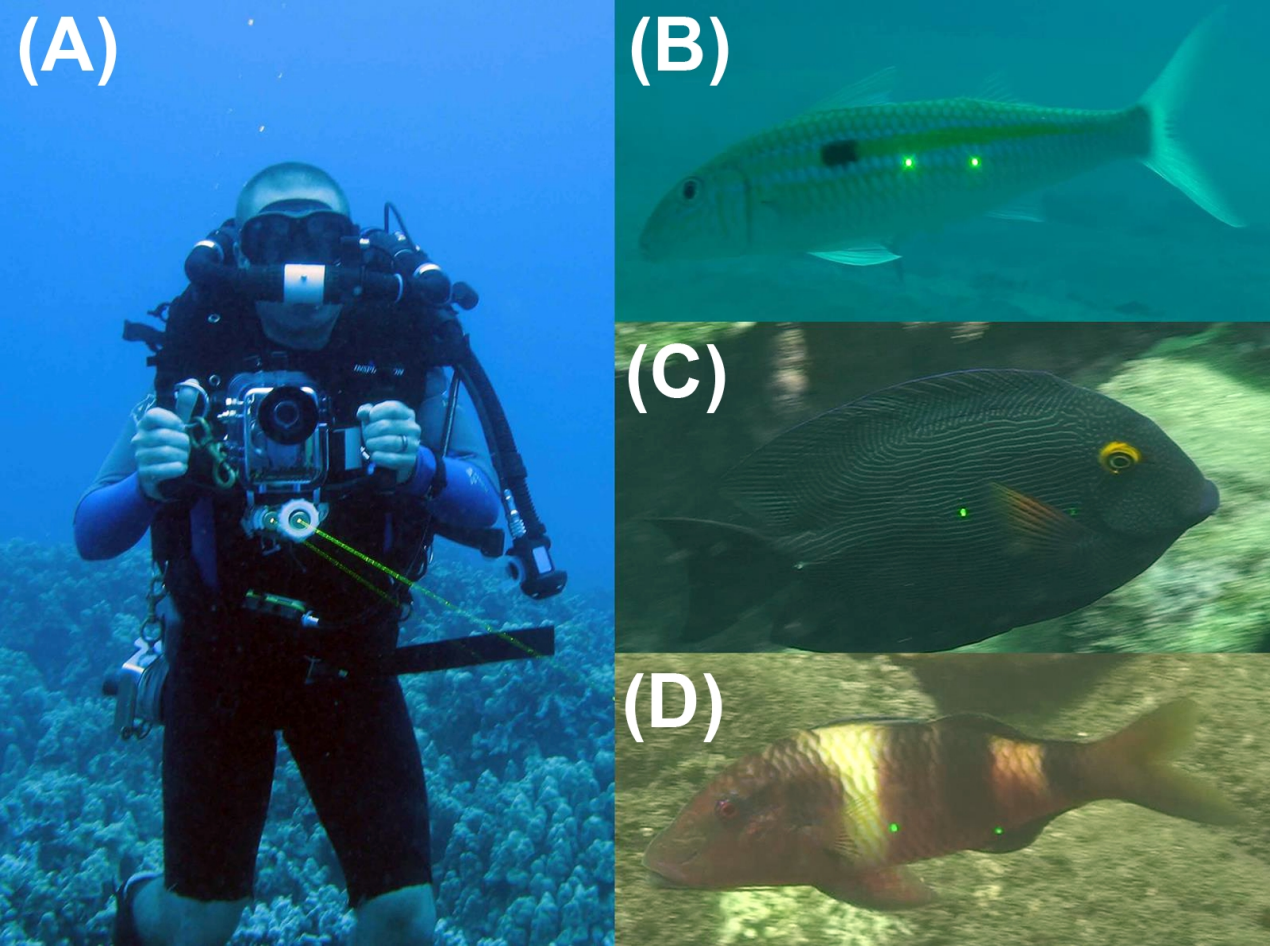
Ross Langston demonstrating the videogrammetry technique for estimating fish sizes. A video camera fitted with parallel lasers (A); superimposes a measurement scale on target fish (B–D). Photos: H Bolick, K Longenecker and R Langston.

Colonies of *Leptoseris* were stained *in situ* using Alizarin Red for subsequent harvesting to determine growth rates (Fig. 5). Additional test colonies of *Leptoseris* were sent to collaborators at the Woods Hole Oceanographic Institution for computerized axial tomography (CT) scanning, ^14^C and U/Th (Uranium–thorium) dating and elemental ratio analyses to determine growth rates. Three fish species exploited on shallow reefs and reported from MCEs, *Centropyge potteri* (Jordan & Metz 1912), *Ctenochaetus strigosus* (Bennett 1828), and *Parupeneus multifasciatus* (Quoy & Gaimard 1825), were selected to compare with existing estimates of production and reproductive output in shallow habitats. We collected specimens to describe length-weight and length-fecundity relationships, growth, and size-specific sex ratios; and to estimate size-at-maturity for mesophotic populations. Laser-videogrammetry surveys (Fig. 6) were used to estimate densities and size structure of target species encountered during the belt transects, based on a high-definition video camera fitted with parallel laser pointers. We then reviewed the video and captured still frames when an individual was oriented perpendicular to the laser beam axes and both lasers appeared on the fish. Because the beams are parallel, the lasers superimpose a reference scale on the side of the fish, allowing length estimates by solving for equivalent ratios. Results of life-history analysis and laser-videogrammetry surveys were incorporated into a modified Ricker production model to estimate annual biomass production and reproductive output for mesophotic populations.

### Broad trophic characterizations

To determine the trophic level of key food web components and functional groups, we conducted stomach content and carbon and nitrogen stable isotopic analyses of reef fishes from 45 species, 30 genera and 18 families (Bradley et al., in press). Further isotope analysis was performed on 24 selected species from seven families without stomach content analysis (Papastamatiou et al., 2015). We also used compound-specific isotope analysis of amino acids to estimate trophic positions of fishes (McClelland & Montoya, 2002; McClelland, Holl & Montoya, 2003; Pakhomov et al., 2004; McCarthy et al., 2007; Popp et al., 2007; Hannides et al., 2009; Hannides et al., 2013) using the difference in *δ*^15^N values of trophic and source amino acids for trophic position calculation (Chikaraishi et al., 2009; Bradley et al., 2015). Additional isotopic analyses were performed on Galapagos sharks [*Carcharhinus galapagensis* (Snodgrass & Heller 1905)] and giant trevally [*Caranx ignobilis* (Forsskål 1775)] in the NWHI (Papastamatiou et al., 2015).

**Figure 7:**
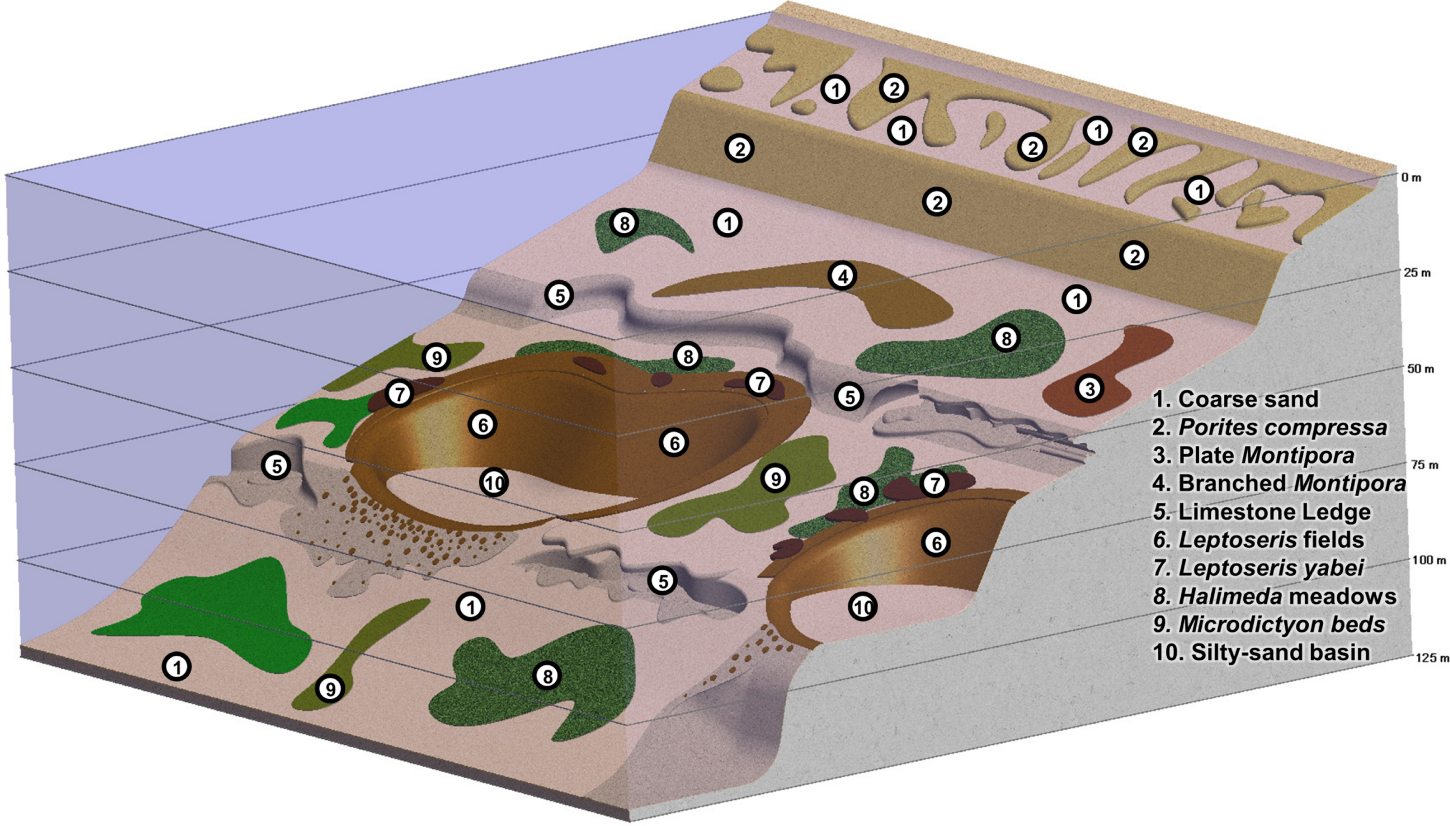
Generalized diagram of major components of MCEs in the ‘Au‘au Channel, Hawaiian Islands. Illustration by RL Pyle.

## Results and Discussion

Our intention in this work is to provide a broad characterization of MCEs across the Hawaiian Archipelago based on more than two decades of interdisciplinary and collaborative research, with emphasis on a seven-year effort to document MCEs in Hawaii largely funded by NOAA. Some portions of this overall research have already been published elsewhere, and others are presented for the first time herein. For purposes of clarity and cohesiveness, we present both novel and previously published information together. When information has previously been published, we provide appropriate literature citations, and when information is presented for the first time, we indicate it as such.

Our investigations revealed that MCEs throughout the Hawaiian Archipelago can be broadly categorized into several distinct habitat types (Fig. 7). The shallowest portions of MCEs (30–50 m) are characterized by a few of the coral species found on shallow Hawaiian reefs, in particular *Montipora capitata* Dana 1846, *Pocillopora meandrina* Dana 1846, *Pocillopora damicornis* (Linnaeus 1758) and *Porites lobata* Dana 1846. At depths of ∼40–75 m, expanses of low relief “carpets” of branching *M. capitata* are found overlying sediment fields, switching to a plate-like or laminar morphology on ledges and rocky slopes (Rooney et al., 2010). All MCE depths had large *Halimeda* spp. meadows and other dominant macroalgal communities over both hard and soft substrates. Although these macroalgal communities generally did not comprise major habitats for large-bodied fishes in the MHI (either in MCE depths, or in shallower areas), endemic reef-associated fishes were found in macroalgal (*Microdicyton* spp.) beds at MCE depths in the NWHI (Kane, Kosaki & Wagner, 2014). Throughout the archipelago, undercut limestone ledges with small caves and other features (the remnants of ancient shorelines) (Fletcher & Sherman, 1995) represent the dominant MCE habitat type at depths of 50–60 m, 80–90 m, and 110–120 m. In certain areas, particularly the ‘Au‘au Channel off Maui and off southeastern Kaua‘i, near-100% *Leptoseris* coral cover extends for tens of km^2^ at 70–90 m. This habitat type, one of the primary subjects of our investigations, represents the most spatially extensive MCE environment documented to date (Costa et al., 2015).

These MCE habitats are often not in close proximity to each other, but separated by vast areas of sand lacking any rocky reef structure. Some of these sandy areas are characterized by meadows of *Halimeda kanaloana* (Maui), *Avrainvillea* sp. and/or *Udotea* sp. (west and south O‘ahu), while others are devoid of organisms associated with coral-reef ecosystems (i.e., non-MCE habitat within MCE depth ranges). Another MCE habitat within the Hawaiian Archipelago that was not a primary subject of investigation for this work, but for which we have extensive qualitative observations, are the steep slopes and drop-offs characteristic of the island of Hawai‘i (i.e., the “Big Island”), especially at the southeastern end of the archipelago. This habitat is dominated by basaltic rock (rather than the coral and limestone, which dominate MCE habitat throughout the rest of the archipelago). Finally, one MCE habitat notably absent from the Hawaiian Archipelago, but prevalent throughout most of the tropical Indo-Pacific, is steep limestone drop-offs, which often extend more or less continuously from shallow-reef depths down to MCE depths and beyond.

In the sections that follow, we highlight and summarize the most salient aspects of MCEs throughout the Hawaiian Archipelago. In particular, we compare and contrast patterns across different MCE habitats, different parts of the archipelago, and among different taxa, as well as emphasize both commonalities and differences among these patterns.

### Geophysical habitat characterization

#### General habitat characterization

MCE habitats in different parts of the archipelago were characterized by contrasting geophysical structures and bathymetric profiles. The general bathymetry of MCEs throughout most of the archipelago (except the island of Hawai‘i, which was not a primary study site) is characterized primarily by gradually sloping flat substrate with occasional rocky outcrops of both volcanic and carbonate material. In most areas, the gradually sloping bottom was interrupted by bathymetric discontinuities at approximately 50–60 m, 80–90 m, and 110–120 m depths (the 80–90 m discontinuity is buried in sand throughout most of the NWHI, except for a small exposed area near Pearl and Hermes Atoll). These discontinuities were typically continuous, rocky undercut limestone ledges or steep sandy or limestone slopes parallel to shore. In some locations, such as within the ‘Au‘au Channel, these discontinuities were the result of karstification (Fig. 2). MCE habitats identified within flat-bottom areas (i.e., between discontinuities) included macroalgal meadows, macroalgal beds, and (especially in the 40–75 m range) expansive low relief beds of interlocking branching colonies, or laminar tiers of *Montipora* spp. Gradually sloping, flat-bottom areas were also commonly surfaced by sand, gravel, rhodoliths and pavement, with very little coral cover. Corals were more common on exposed rock surfaces along rock ledges and outcrops. In contrast to most sites in the Hawaiian Archipelago, the 80–90 m discontinuity within the ‘Au‘au Channel included very few exposed rocky areas at MCE depths, except along very steep walls and in a few areas otherwise dominated by *Leptoseris* spp.

**Figure 8:**
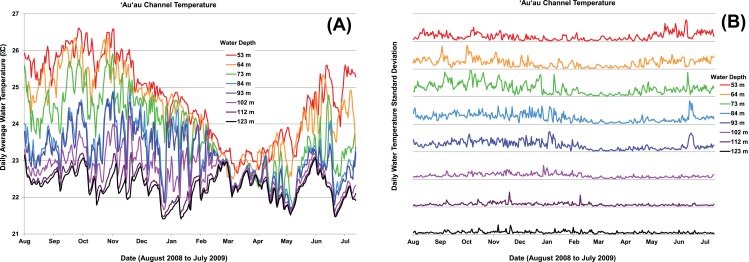
Temperature data from “John” and “Frank” moorings. Temperature data from “John” and “Frank” moorings, comparing seasonal and daily fluctuations in water temperature at each of eight different depths off the ‘Au‘au Channel from August 2008 to July 2009. Graphs represent the average daily temperature (A) and the daily standard deviation (SD) (B) at each depth. The thin black line below each depth trace in (B) represents SD = 0, and the thin black line above represents SD = 1; the greater the distance of the color line from the black line below (SD = 0), the more dynamic the daily temperature. SD is based on *n* = 72 temperature values/day for data recorded at 84 and 123 m, and *n* = 36 for other depths.

#### Geophysical factors

One of the most important geophysical characteristics of MCEs in Hawai‘i is water clarity. Within the ‘Au‘au Channel, MCEs were found to occur in areas offshore with very clear water, with a diffuse attenuation coefficient (*K*_*o*_) of 0.041 ± 0.001 m^−1^. In comparison, nearby areas inshore of west Maui had higher attenuation coefficients (and thus more turbid water), ranging from 0.107 m^−1^ at 10 m depth to 0.073 m^−1^ at 30 m depth (Spalding, 2012). The average percent surface irradiance (SI) and irradiance values (PAR) at depth were 10% SI at 34 m (245 ± 15 SE μE m^−2^ s^−1^), 1% SI at 90 m (25 ± 3 SE μE m^−2^ s^−1^), and 0.1% at 147 m (2.5 ± 0.4 SE μE m^−2^ s^−1^). The 1% SI is often referred to as the compensation point, where photosynthesis equals respiration; above this point, there is net photosynthesis and production of organic matter; below this point, there is net consumption of organic material, and respiration exceeds photosynthesis (Kirk, 2011). In general, areas with the clearest water also supported the richest and most expansive MCEs.

**Figure 9:**
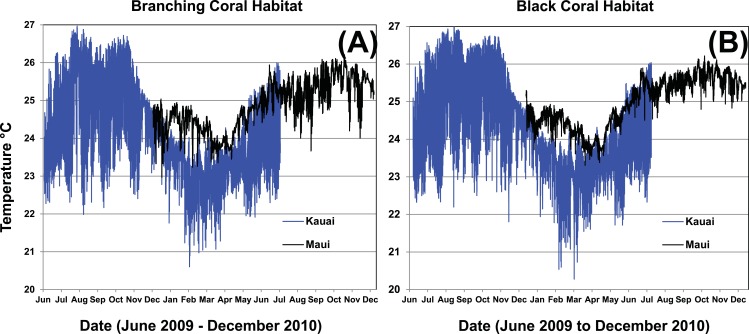
One-year temperature profile in two MCE habitat types at Kaua‘i and Maui. Branching coral (*Montipora*; A) habitat was at approximately 57 m and black coral (*Antipathes*, B) habitat was at 34 to 62 m.

Water temperatures in the Au‘au Channel from August 2008 through July 2009 ranged from just below 21°C to just over 26.5°C throughout the water column over the time period sampled. A seasonal temperature cycle was apparent throughout the water column, with warmest temperatures from September to November, and coolest from February to May. The temperature was consistently 2–3°C cooler at the deep end of the sampled depth range (Fig. 8A), with less short-term variability and less seasonal fluctuation. Water temperatures were logged within three depth ranges: shallow (53 and 64 m), middle (73, 84, and 93 m), and deep (102, 112, and 123 m). Water temperatures at the deepest depths were the most stable on a daily basis, whereas temperatures at the middle depths were the most dynamic. The shallowest depths were intermediate in terms of daily thermal stability (Fig. 8B). Relatively large (1–2°C) short-term (1 day) temperature excursions occurred at 50–75 m, however, temperatures were very consistent on this time scale at the deep site (Fig. 8B). The most dynamic temperatures for all but the deepest three depths, corresponded with the warmest months (September to November), and the most thermally stable months were the coolest (February to May). We were unable to determine whether MCEs are influenced by tidally-correlated vertical thermocline shifts. The establishment of moorings at the deep extreme of the MCE range would provide useful comparative data to test for a tidal effect, versus bathymetric forcing. In March of 2009, the “Tele 1” mooring slid down slope, causing the temperature sensor to change depth, so data from this mooring were not included in the analyses. A comparison of temperatures between Maui and Kaua‘i at 34–62 m showed that both islands had a seasonal trend, but Kaua‘i had a higher daily and seasonal fluctuation than Maui (Fig. 9).

**Figure 10:**
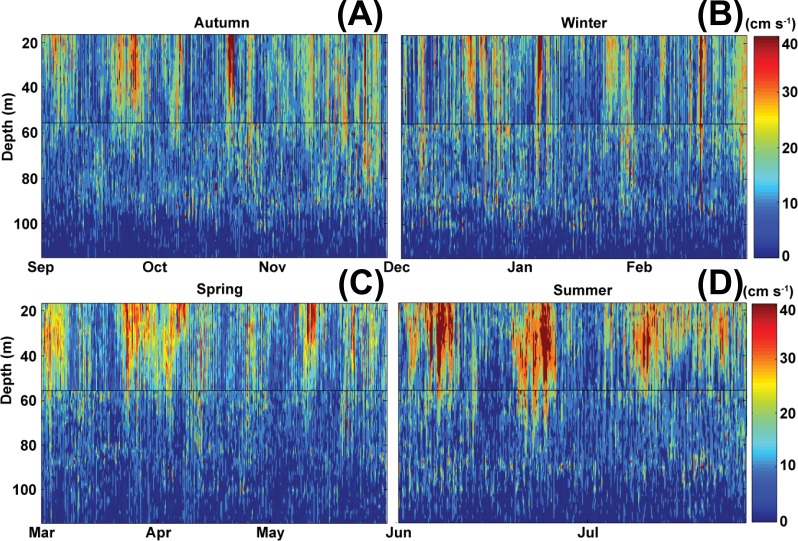
Current magnitude profiles. Sontek 250 kHz Acoustic Doppler Profiler profiles of current magnitude in cm s^−1^, with overlap shown in the black line between the deeper Frank mooring and shallower John mooring. Broken down by seasons to show detail (A, Autum; B, Winter; C, Spring; D, Summer).

Acoustic profiler analysis indicated that the current magnitude at 70–90 m where corals were most abundant fluctuated between 10–15 cm s^−1^ with sporadic, brief pulses >25 cm s^−1^, with a clear pulsing (strengthening and weakening) that corresponded with direction changes on a tidal frequency (Fig. 10). At greater depths, the flow was almost stagnant with little tidal signal and variable direction. These results are in stark contrast to the higher magnitude currents (up to more than 40 cm s^−1^) that occur at shallow depths subject to daily tidally forced flows (Fig. 10). Although there were clear differences in flow rates observed at MCE depths, the observed direction of flow was highly variable and difficult to attribute to tidal or wind driven processes. Greater resolution in sampling would be needed to determine the relationship between flow direction and reef orientation for MCE habitats.

### Biodiversity patterns

#### Species diversity

Seventy-two species of frondose macroalgae were identified based on morphological characteristics from MCEs in the MHI, including 29 Chlorophyta, 31 Rhodophyta, and 12 Phaeophyceae. Estimates of macroalgal diversity are likely conservative because of taxonomic limitations regarding morphological identifications. For instance, large green algal “sea lettuce” blades from MCEs were all identified morphologically as “*Ulva lactuca*.” However, recent molecular analyses revealed that these specimens represent four new species belonging to the genera *Ulva* and *Umbraulva*, which cannot be identified using morphological characters alone (Spalding et al., 2016). Nevertheless, the methods used were similar to current taxonomic treatments in Hawai‘i (Abbott, 1999; Abbott & Huisman, 2003; Huisman, Abbott & Smith, 2007) allowing for comparisons with the better-known shallow-water flora. Macroalgal communities were found in discrete patches (separated by sand or other benthic habitats) at all MCE depths in the MHI. Examples include expansive meadows of *Halimeda kanaloana* Vroom in sand, beds of *Halimeda distorta* (Yamada) Hillis-Colinvaux over hard substrates, as well as monospecific beds of *Distromium* spp., *Dictyopteris* spp., *Microdictyon* spp., *Caulerpa* spp., and mixed assemblages of other macroalgal species (Spalding, 2012). These MCE macroalgal assemblages are abundant, diverse, and spatially heterogeneous with complex distributional patterns, contributing to heterogeneous structural complexity. In contrast, MCE macroalgal communities in the NWHI tended to be dominated by beds of *Microdictyon* spp., although ROV video around the banks of the NWHI also show regions of *Sargassum* or *Dictyopteris* species (Parrish & Boland, 2004) (collections not yet available for verification).

Of the approximately fifty species of scleractinian corals known from the Hawaiian Islands (Hoover, 1998), ten were recorded by this study from MCEs in the MHI. Three of these—*Pocillopora damicornis*, *P. meandrina*, *Porites lobata*, and *Montipora capitata*—are common species on adjacent shallow reefs. A previously published phylogeny resolved six *Leptoseris* species in Hawai‘i: *Leptoseris hawaiiensis* Vaughan 1907, *L. papyracea* (Dana 1846), *L. scabra* Vaughan 1907, *L. tubilifera* Vaughan 1907, *L. yabei* (Pillai & Scheer, 1976), and a putative undescribed species, “*Leptoseris* sp. 1” (Luck et al., 2013; Pochon et al., 2015). However, the reliability of coral phylogenies has been challenged due to limited genetic variation (Sinniger, Reimer & Pawlowski, 2010). In addition to scleractinian corals, eight antipatharian coral species were documented from MCE depths through work associated with this study and published previously (Wagner et al., 2010; Wagner et al., 2011; Wagner, 2015a; Wagner, 2015b).

**Figure 11:**
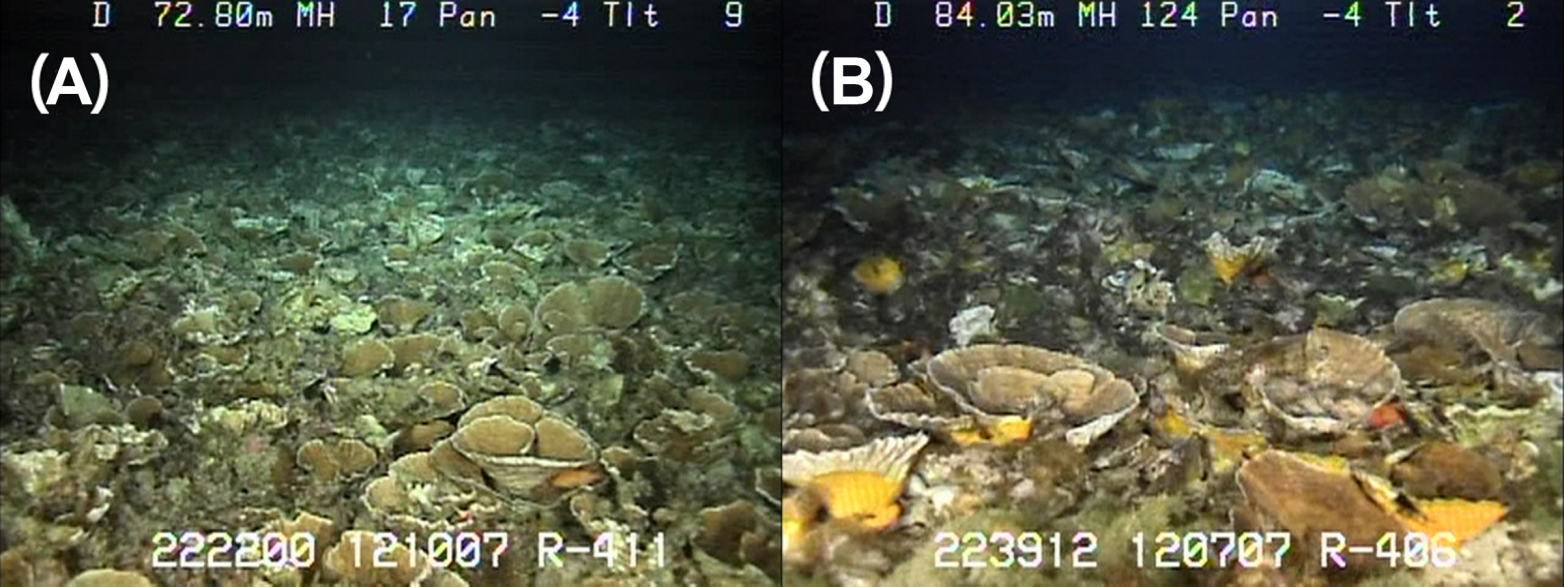
Comparison of *Leptoseris*-dominanted MCE habitats. (A) Kaua‘i and (B) Maui, showing the close similarity in general structure.

One of the goals of this research was to identify key parameters that might determine the presence and distribution of MCE habitats elsewhere in Hawaiian waters through the development of a spatial model. This portion of our study has been published previously (Costa et al., 2015), but in summary, depth, distance from shore, euphotic depth and sea surface temperature were identified as the four most influential predictor variables for partitioning habitats among the three genera of corals included in the modeling exercise (*Leptoseris*, *Montipora*, and *Porites*). Costa et al. (2015) found that for corals that occur in the shallower depth (50 m) of MCEs, hard substrate is necessary, but not sufficient, for colonization. It is less certain whether hard substrate is necessary at greater depths, where some of the *Leptoseris* beds were found in a density of three or more layers of coral plates deep. Whether this is due to accretion on a hard substrate or a stable soft bottom needs further examination. Additional details of the methods and results from this portion of the study are available in Costa et al. (2015). Extensive *Leoptoseris*-dominated MCEs with very similar structure, depth and species composition have been identified in two MHI regions: the ‘Au‘au Channel, and off southeastern Kaua‘i (Fig. 11). Although no other *Leoptoseris*-dominated MCEs have yet been located within the Hawaiian Archipelago, similar MCE habitats may exist elsewhere in the MHI.

**Table 2:** Comparison of fish assemblages associated with black coral beds of two Main Hawaiian Islands, Kaua‘i and Maui. Feeding guild percentages from number of fish in the feeding guild compared to total number of fish observed in the island’s black coral bed.

	Maui	Kaua‘i
Total fish observed	2,080	1,322
Number of species observed	60	52
Herbivore	7%	5%
Planktivore	68%	67%
Omnivore	1%	1%
Benthic Carnivore	23%	26%
Piscivore	1%	1%

In addition to corals, nearly 200 specimens from among eight phyla of marine invertebrates (Foraminifera, Porifera, Bryozoa, Annelida, Arthropoda, Cnidaria, Ophiuroidea, and the subphylum Urochordata) were collected from MCEs in the MHI. Unfortunately, many of these groups are poorly known taxonomically, and nearly three quarters of these specimens remain unidentified.

Qualitative comparisons of fish assemblages associated with black coral beds are available for two main islands: Kaua‘i (this study) and Maui (Boland & Parrish, 2005). Both were similar in species number; the Maui survey recorded 60 species and Kaua‘i had 52 (Table 2). A Wilcoxon Signed Rank Test showed no significant difference (*P* = 0.100, *W* = − 1.647) in species abundance. When all fish were categorized by feeding guilds, both had similar distributions. Both were dominated by planktivores with Maui at 68% and Kaua‘i at 67%, with the next largest group being benthic carnivores at 23% and 26%, respectively (Table 2). By comparison, black coral MCEs of the Mid-Atlantic Ridge host 33% planktivores and 9% benthic carnivores (Rosa et al., 2016).

**Figure 12:**
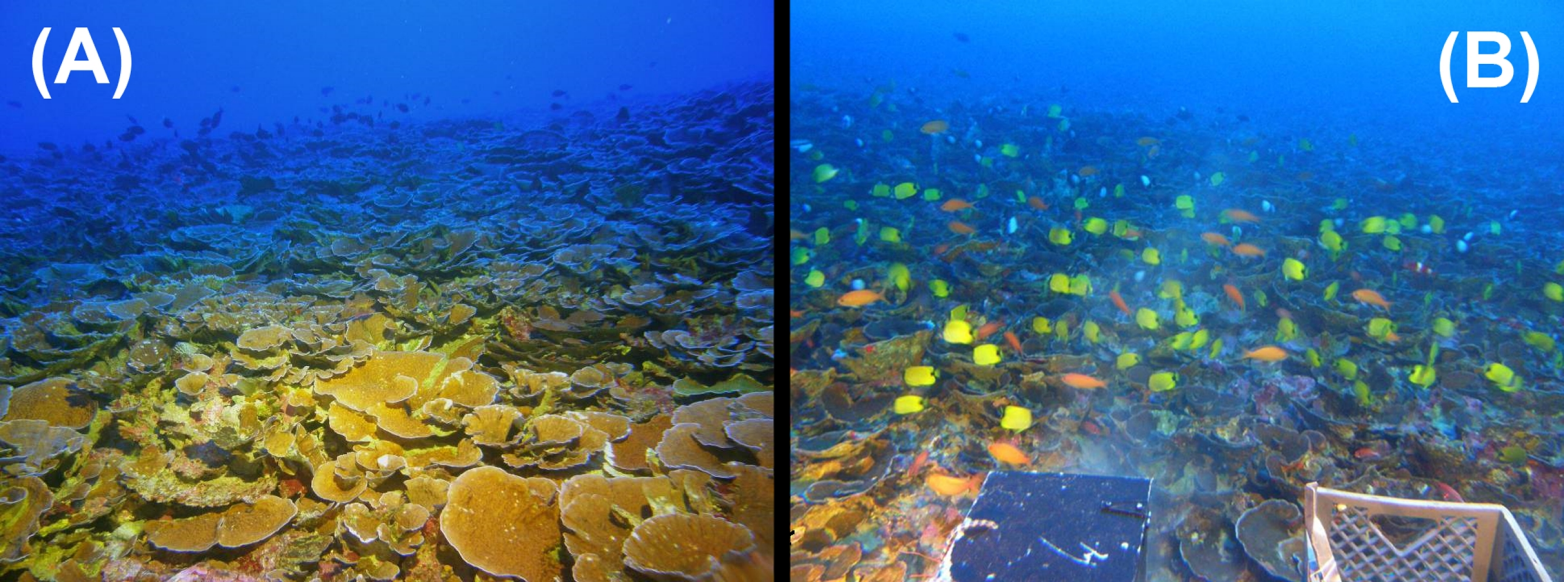
Heterogeneous reef fish distribution on *Leptoseris* reefs in the ‘Au‘au Channel. Reef fish distribution on *Leptoseris* reefs in the ‘Au‘au Channel was heterogeneous, with large areas nearly devoid of fishes (A) punctuated with areas of high fish diversity and abundance (B). The fishes seen in the distance in (A) represent a separate localized area of high abundance. All but two of the fishes visible in (B) belong to endemic species (Endemics: *Chaetodon miliaris, Pseudanthias thompsoni, Sargocentron diadema, Dascyllus albisella, Holacanthus arcuatus, Centropyge potteri;* Non-endemic: *Forcipiger flavissimus, Parupeneus multifasciatus*). Photos: HURL.

Within the ‘Au‘au Channel site, both divers and submersible observers made repeated anecdotal observations of highly heterogeneous fish diversity and abundance over *Leptoseris* beds with similar morphology and abundance. Some areas were almost devoid of fishes (Fig. 12A), whereas others harbored high levels of both diversity and abundance (Fig. 12B). No transect data were performed to quantify this preliminary observation, and we can think of no obvious reason why the pattern might exist. However, we feel this observation is interesting enough, and was made consistently enough, to justify noting here in the hope of prompting future research.

Elsewhere in the Pacific, MCEs harbor high numbers of species new to science (Pyle, 2000; Rowley, 2014). The fish fauna of Hawai‘i has been better documented than any other location in the tropical insular Pacific, so new species were not expected. However, at least four undescribed species of fishes have been collected on MCEs in Hawai‘i, including a highly conspicuous butterflyfish (*Prognathodes*) and three other less conspicuous species (*Scorpaenopsis*, *Suezichthys* and *Tosanoides*). These have been determined by experts in the respective taxonomic groups to be undescribed, and are in various stages of formal description. At least one putative new species of scleractinian coral (*Leptoseris*) has been identified (as noted above), and one putative new species and one new record of Antipatharian corals for the Hawaiian Archipelago have been recorded (Wagner et al., 2011; Opresko et al., 2012; Wagner, 2015a). Undescribed macroalgae will require molecular characterizations that will likely increase the number and diversity of recognized species. Likewise, the taxonomy of many groups of marine invertebrates is poorly known (see Hurley et al. 2016), and many of the unidentified specimens of invertebrates may prove to be new species, once subjected to the same taxonomic scrutiny by appropriate experts that the aforementioned fishes, corals and algae have undergone. Sponge taxonomists have indicated numerous undescribed species and genera from among MCE collections. This is likely the case for the other phyla as well, especially within polychaetes, small crustaceans, and tunicates.

#### Depth zonation

The preponderance of new species within MCE habitats reinforces the observation that the species inhabiting deeper MCEs are generally different from those inhabiting shallow reefs. Overall, diversity was lower on MCEs than on nearby shallow reef habitats. This pattern was consistent for macroalgae, corals, macroinvertebrates and fishes. However, within MCE habitats, different taxonomic groups showed different patterns of diversity.

**Figure 13:**
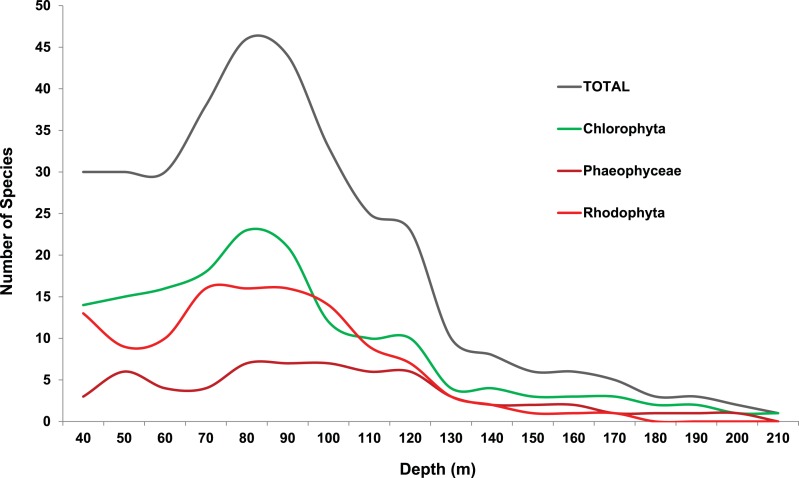
Total number of macroalgal species (over all sites combined) found at each depth surveyed. Depth of occurrence is based upon collections and visual observations when species level identifications were verified. Shallower depths (40–60 m) were collected by mixed-gas divers while depths ≥70 m were collected by submersibles. See Spalding (2012) for collection locations. Data are included in the “AlgaeData” (Supplemental Information 2; Tab 1) worksheet of the Raw Data file.

Survey results for macroalgae show more species at 70–100 m compared to 40–60 m, with the most distinctive changes in diversity (i.e., the most substantial changes in total number of species at each depth interval) occurring at 80–90 m and 110–120 m depths (Fig. 13). These depths corresponded to ∼3% and 0.5% of SI, respectively, and included depths where large changes in seasonal thermoclines were observed. The water column at most sites was characterized by high clarity and deep penetration of irradiance (10 µmol m^−2^ s^−1^ at 110 m depths), although sedimentation from terrigenous sources appeared to reduce macroalgal abundance at a few sites.

A similar pattern may exist for scleractinian corals. Three species of corals were found at 30–50 m (*M. capitata*, *P. damicornis* and *P. lobata*). *P. damicornis* and *P. lobata* were only seen at the shallowest MCEs (<50 m), while *M*. *capitata* occurred at greater depths (50–80 m) (Rooney et al., 2010). At these greater depths, *M. capitata* was most commonly observed in a branching morphology that formed low-relief reefs carpeting tens of km^2^ of sea-floor off the west coast of Maui. Similar reefs have been observed off Kaua‘i and Ni‘ihau, although a plate-like morphology is dominant around O‘ahu. At greater depths, the dominant corals are within the genus *Leptoseris*. Starting at a depth of ∼65 m, *Leptoseris* corals were most commonly encountered, becoming the dominant corals at depths below ∼75 m, and continuing in high abundance down to 130 m, with solitary colonies at depths in excess of 150 m (Rooney et al., 2010). Of these, recent evidence indicates that *L. hawaiiensis* was found exclusively at depths below 115–125 m (Pochon et al., 2015). The latter study also investigated endosymbiotic dinoflagellate *Symbiodinium* and resolved three unambiguous haplotypes in clade C, with one haplotype exclusively found at the lower MCE depth extremes (95–125 m) (Pochon et al., 2015). These patterns of host–symbiont depth specialization indicate limited connectivity between shallower and deeper portions of MCEs, and suggest that niche specialization plays a critical role in the host–symbiont evolution of corals at MCE depth extremes.

Invertebrate identifications completed thus far indicate that many species from various phyla are deep-water specialists, such as the polychaete *Eunice nicidioformis* (Treadwell 1906), which was originally described from a specimen collected at 200–300 m in the Hawaiian Islands. Hurley et al. (2016) observed strong zonation of brachyuran crab species by depth off O‘ahu, and this trend is likely to extend to all invertebrate phyla. Remarkably few amphipods were found, and most were parasitic or inquiline species (Longenecker & Bolick, 2007); possibly an artifact of sampling with submersibles (i.e., free-living species may have been swept off during ascent).

**Figure 14:**
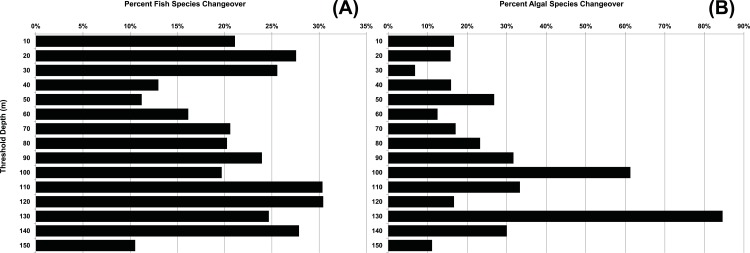
Fish and macroalgal species changeover at 10-m depth intervals. The degree of fish (*n* = 445) changeover (A) and macroalgal (*n* = 72) species changeover (B) at 10-m depth intervals. Values of each bar represent the number of species with a maximum known depth limit within 10 m above each depth interval plus the number of species with a minimum known depth limit within 10 m below each interval, expressed as a percentage of the total species present at the interval. A high value indicates a more substantial break, and a low value represents a less substantial break. Data are included in the “AlgaeData” ((Supplemental Information 2; Tab 1)) and “FishData” (Supplemental Information 2; Tab 2) worksheets of the Raw Data file.

The pattern of depth stratification was much less apparent among fishes. Depth ranges of all reef-associated fish species known to occur at depths of less than 200 m (*n* = 445) were obtained through this study and from historical literature, and are included in the “FishData” (Supplemental Information 2; Tab 2) worksheet of the Raw Data file. Among species recorded at depths greater than 30 m (*n* = 346), 87% (*n* = 302) also occur at shallower depths (i.e., only 12% (*n* = 44) of fishes recorded from MCEs are restricted to MCEs). In the Northwestern Hawaiian Islands, Fukunaga et al. (2016) found that fish assemblages at mesophotic depths (27–67 m) had higher densities of planktivores and lower densities of herbivores than on comparable shallow reef-fish assemblages between 1 and 27 m. It has been suggested that there may be a consistent and relatively sharp faunal break at around 60 m (Slattery & Lesser, 2012). An analysis of *beta*-diversity among fishes in the Red Sea found that the rate of species turnover increased with depth (Brokovich et al., 2008); however, this study only extended to 65 m, the shallowest portion of MCEs. Moreover, traditional approaches to quantifying beta-diversity changeover are designed to measure presence/absence data for multiple discrete zones, and would require multiple replicate transects at multiple depth zones across many different habitats and geographic locations (i.e., potentially thousands of transects) to adequately characterize species transition patterns. Instead, the question of where the largest and most substantial species assemblage transitions occur can be addressed by a more holistic approach to known species depth ranges (see Supplemental Information 1). Figure 14A, which summarizes data included in the “FishData” (Supplemental Information 2; Tab 2) worksheet of the Raw Data file, reveals that the most substantial faunal transitions in fishes occur in the range of 10–30 m and 110–140 m, and the least substantial transitions occur in the range of 40–60 m, with moderate transitions in the range of 70–100 m. The most substantial floral transitions (from data included in the “AlgaeData” (Supplemental Information 2; Tab 1) worksheet of the Raw Data file) occur between 90–110 m and at 130 m depths (Fig. 14B), indicating that 60 m is not a significant transition for species changeover in macroalgae. Brachyuran crabs show the strongest transitions between 60 and 90 m (Hurley et al., 2016). The diversity of coral species is insufficient to allow the application of this type of analysis; however, applying this method to other taxa and in other regions should provide insight into whether floral and faunal breaks are consistent on a broader taxonomic and geographic scale.

#### Endemism

Our findings support previous reports of higher rates of endemism (species that occur only within the Hawaiian Islands) among fishes on MCEs (Pyle, 1996b; Pyle, 2000; Kane, Kosaki & Wagner, 2014). Among 259 species of fishes recorded on MCEs across the Hawaiian Archipelago, 70 (27%) are endemic (inclusive of Johnston Atoll), considerably higher than the 20.5% of endemic fishes across all reef and shore fishes reported for the Hawaiian Archipelago (Randall, 1998). However, with more careful analysis, the trend of increasing endemism with increasing depth within MCEs is even stronger.

**Figure 15:**
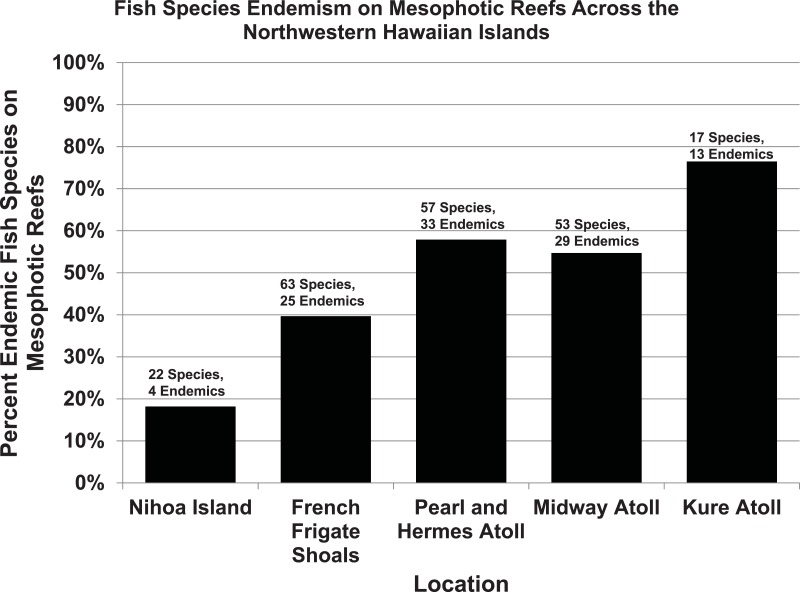
Proportion of endemic reef fish species in mesophotic fish communities of the NWHI.

**Figure 16:**
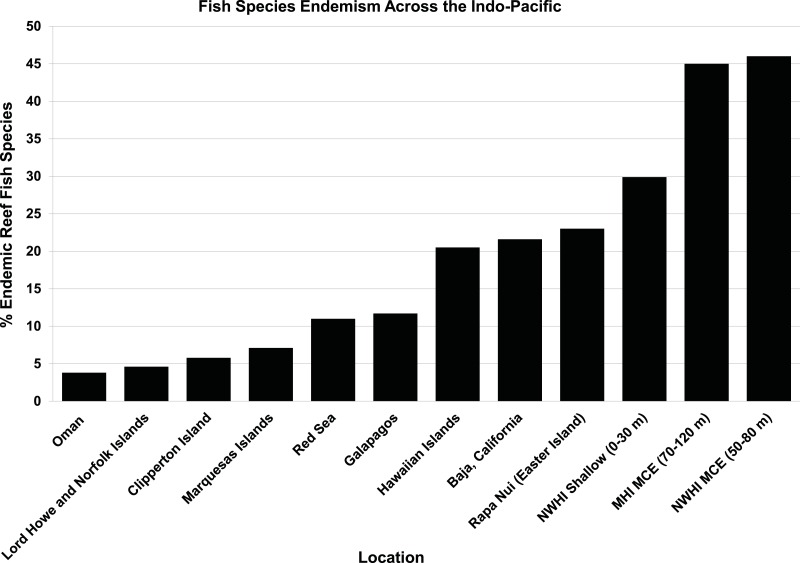
Proportion of endemic coral reef fish species across the tropical Indo-Pacific by island/region. Sources: Randall, 1998; Moura & Sazima, 2000; Allen, 2008; Floeter et al., 2008. Kane, Kosaki & Wagner, 2014; this study.

Based on our surveys, the rate of endemism among reef fishes found exclusively shallower than 30 m (*n* = 126) was 17%, and the rate of endemism among reef fishes found exclusively deeper than 30 m (*n* = 42) is 43%. The rate of endemism remained roughly the same (16–17%) for fishes found only shallower than 40 to 80 m depths (in 10-m depth increments), but changed to 44% for fishes found only deeper than 40 m, 41% below 50 m, 50% below 60 m, and 51% for fishes found only deeper than 70 m. This trend appears to be restricted to fishes inhabiting MCEs (rather than a general trend of increasing endemism with increasing depth), because among fishes restricted to depths greater than 150 m, the rate of endemism is 14% (Mundy, 2005). The proportion of endemic fish species increases even further with increasing latitude across the Archipelago. At the northwestern-most atolls, endemism among MCE fishes reaches 76% (Fig. 15). This represents one of the highest rates of endemism reported for any marine ecosystem, which could be due to cooler water temperatures limiting the northward distribution of tropical species (Kane, Kosaki & Wagner, 2014). Figure 16 shows a comparison of overall rates of endemism among fishes in both MCEs and shallow reefs of the NWHI and ‘Au‘au Channel, against general rates of endemism (mostly biased toward shallow reefs) for the tropical Indo-Pacific and Eastern Pacific. The trend towards elevated endemism is even stronger when relative abundance is taken into consideration. Not only are more endemic species found on MCEs, but the endemics also tend to be the most abundant species. At the northernmost end of the NWHI, the relative abundance of endemic reef fishes exceeds 92% (Kane, Kosaki & Wagner, 2014), and even reach 100% in some places (Kosaki et al., 2016). This pattern is also evident in the MHI, as illustrated by Fig. 12B (taken at 90 m in the ‘Au‘au Channel) in which all but two of the hundreds of fishes are endemics. This pattern may represent a combination of both depth and latitudinal gradients, and ongoing quantitative surveys throughout the Hawaiian Archipelago should reveal more detailed interpretations of these patterns.

Patterns of endemism among other groups (particularly macroalgae and marine invertebrates) are more difficult to quantify. The full extent of endemism and the broader diversity within the MCE flora can only be determined after molecular studies are conducted and data are gathered from similar MCE habitats elsewhere in the Pacific. Until the unidentified specimens of invertebrates from MCEs are examined by appropriate experts, and comparable sampling is conducted elsewhere in the Pacific, it will not be possible to determine proportions of endemism among marine invertebrates on Hawaiian MCEs.

**Figure 17:**
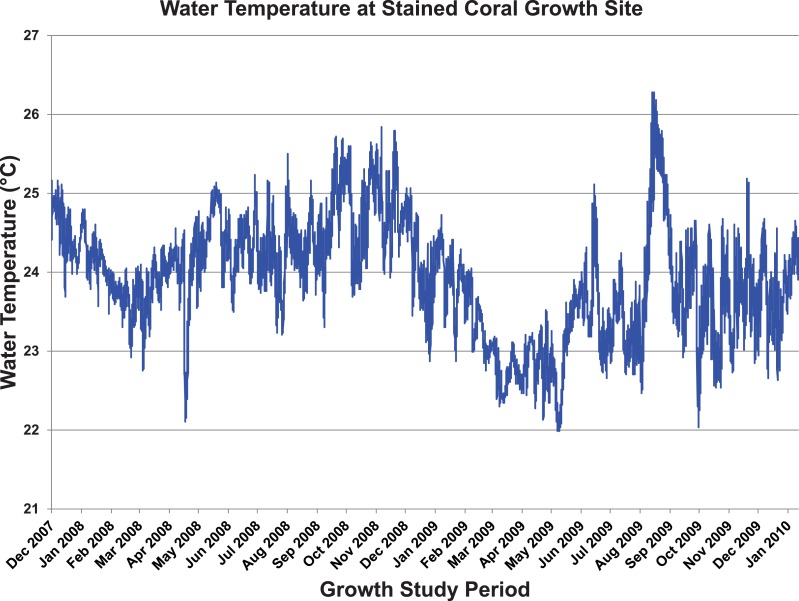
Temperature log from stained coral.

### Population dynamics

We hypothesized that growth rates (the radial extension of the carbonate plate) of *Leptoseris* sp. corals, the dominant benthic organism at depths of 70–150 m in the Main Hawaiian Islands, were similar to the only published growth rate for *L*. *fragilis* from the Red Sea (0.2–0.8 mm yr^−1^) (Fricke, Vareschi & Schlichter, 1987). Because direct measurement via submersible was unlikely to have the precision to measure such rates over the period of our study, colonies were stained for later recovery and analysis. The stained colony recovered from a depth of 83 m had stain appearing only in two marked patches of the skeleton, making it difficult to conclusively describe the complete radial growth of the coral plate.

X-radiographic imaging of entire *Leptoseris* test colonies did not reveal the resolution required to discern banding patterns. Additional colonies examined using CT scanning of the entire plate yielded excellent images, clearly revealing fine banding parallel to the outer edge of the colony. However, Δ14C analysis showed that all samples were younger than the 14C peak from the early 1960s that occurred as a result of nuclear weapons testing. This result indicated that bands are approximately an order of magnitude too closely spaced to be annual, but the cause of the banding remains unclear. The edge adjacent to the stain on the marked colony showed an addition of 12 to 13 new bands but these did not correspond to the two-year seasonal temperature cycle from the data logger deployed when the coral was marked (Fig. 17). This first marked colony indicates some type of monthly banding cycle so the additional colonies stained *in-situ* are an important part of future studies. A series of U/Th dates from eight representative portions of the stained colony indicate a colony age of ∼15 years (Table 3). Although 2-*σ* uncertainty for U/Th dating is generally ±0.1 years, each sub-sample integrates material from several years and different sides of the colony extended further from the center. Also, no sample was collected from the exact center of the colony, but based on the age difference between the two samples nearest the center (LH1, LH2) approximately another 2.2 years was added to the age of sample LH1 to determine a final colony age of ∼14.8 years at the time of recovery. The mean of several radius measurements is 14.9 cm providing a mean growth rate of ∼1 cm yr^−1^, or more than an order of magnitude faster than that reported for *L. fragilis*.

**Table 3:** Ages of *Leptoseris* sp. colony samples based on Uranium/Thorium (U/Th) dating techniques. The colony was marked in December 2007 and sampled January 2010.

Sample	Uranium–Uranium 234/238 activity	Thorium–Uranium 230/238 activity	Initial delta ^238^U	Age (yr) before 2012.75	Estimated sample age (yr)
LH1	1.1467 ± 0.0002	0.000160 ± 7.1 × 10^−7^	146.7 ± 0.23	15.3 ± 0.07	14.8
LH2	1.1470 ± 0.0002	0.000137 ± 7.0 × 10^−7^	147.0 ± 0.19	13.1 ± 0.07	10.4
LH3	1.1470 ± 0.0002	0.000132 ± 9.5 × 10^−7^	147.0 ± 0.17	12.6 ± 0.09	9.9
LH4	1.1469 ± 0.0002	0.000138 ± 1.1 × 10^−6^	146.9 ± 0.23	13.1 ± 0.11	10.4
LH5	1.1469 ± 0.0003	0.000061 ± 5.6 × 10^−7^	146.9 ± 0.27	5.8 ± 0.05	3.1
LH6	1.1469 ± 0.0002	0.000053 ± 3.3 × 10^−7^	146.9 ± 0.18	5.0 ± 0.03	2.3
LH7	1.1471 ± 0.0002	0.000138 ± 8.6 × 10^−7^	147.1 ± 0.24	13.2 ± 0.08	10.5
LH8	1.1468 ± 0.0002	0.000112 ± 7.3 × 10^−7^	146.8 ± 0.18	10.7 ± 0.07	8.0

Logistical constraints associated with working at mesophotic depths severely limited the number of specimens available for life-history analysis (37 *Centropyge potteri*, 33 *Ctenochaetus strigosus*, and 33 *Parupeneus multifasciatus*), and the number of length estimates obtained from laser-videogrammetry surveys (21 *C. potteri*, 28 *C.strigosus*, and 90 *P. multifasciatus*). Thus, the results presented here should be considered preliminary and did not warrant rigorous statistical analysis. Table 4 presents densities, average lengths, and life-history parameters for mesophotic populations from the ‘Au‘au Channel and for previously studied shallow-water populations from across the main Hawaiian Islands. The results were not consistently higher or lower in MCEs compared to shallow reefs. The net effect of these differences, when combined with size-structure data, was predicted by the Ricker model (Everhart & Youngs, 1992), which we modified as described in the detailed ‘Materials and Methods’. The results presented in Table 5 indicate that biomass and egg production estimates were lower at MCE depths, and estimates for shallow depths are at least an order of magnitude higher for all except the egg production of *P. multifasciatus*. The parameters used in the Ricker model interact in complicated ways, making it difficult to determine reasons for the differences between MCEs and shallow depths. Given the admittedly preliminary nature of our results, we are unwilling to speculate on the cause(s) of these differences. Nevertheless, the possibility that biomass production and reproductive output of exploited fish populations are lower in MCEs deserves full consideration in future fishery management and habitat conservation efforts.

### Broad trophic characterizations

**Figure 18:**
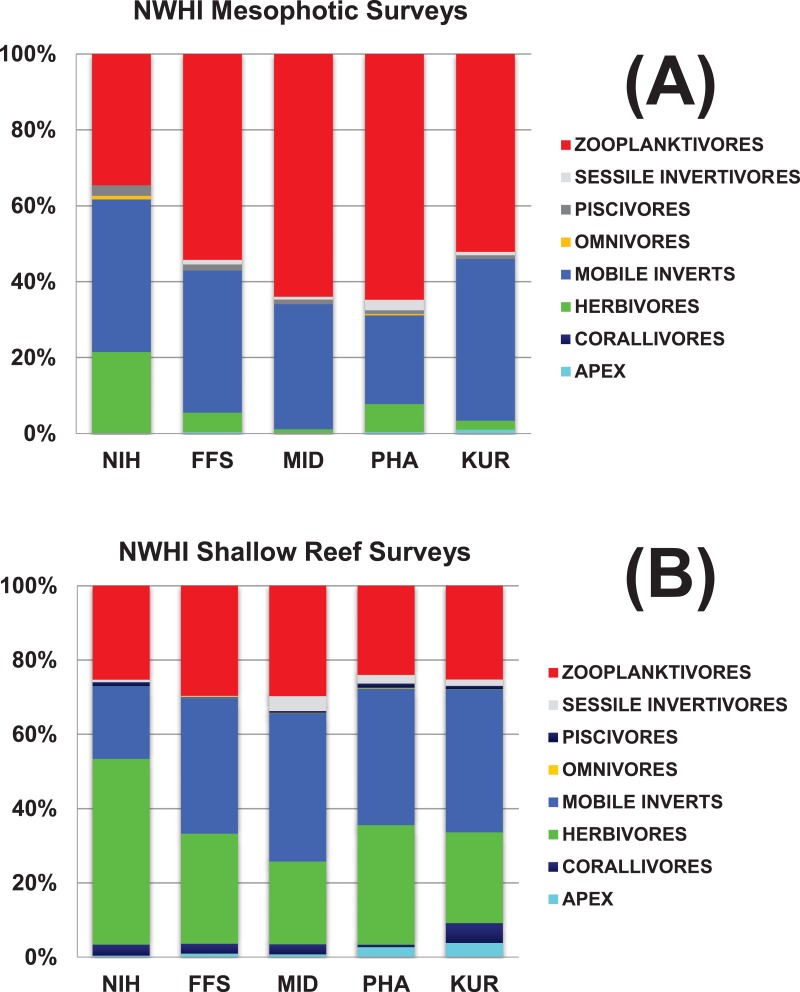
Comparison of fish assemblage trophic structure between shallow and mesophotic reefs in the NWHI. NIH, Nihoa; FFS, French Frigate Shoals; MID, Midway Atoll; PHA, Pearl and Hermes Atoll; KUR, Kure Atoll.

The relative representation of different trophic groups of fishes on shallow reefs and MCEs in the NWHI is illustrated in Fig. 18. Shallow reefs were numerically dominated by herbivores and mobile invertivores, whereas MCE fish communities were numerically dominated by planktivores (See the “NWHIFishTrophic” (Supplemental Information 2; Tab 3) Worksheet of the Raw Data file).

**Table 4:** Life history and population characteristics of exploited fishes at MCE and euphotic depths. Lengths (L) as fork length (FL) or total length (TL) are in mm, weights (W) are in g, time (t) is days, batch fecundity (BF) is number of eggs. MCE columns represent the ‘Au‘au Channel, shallow columns from previous studies throughout the main Hawaiian Islands (Longenecker & Langston, 2008; Langston, Longenecker & Claisse, 2009).

	*C. potteri* (TL)		*C. strigosus* (FL)		*P. multifasciatus* (FL)	
	MCE	Shallow	MCE	Shallow	MCE	Shallow
Density (#/m^2^)	0.0024	0.0120	0.0025	0.0524	0.0287	0.0442
Mean L	77.7	70.0	96.7	99.2	122.5	133.8
L-W	*W* = 4.99⋅10^−5^(*L*)^2.877^	*W* = 2.28⋅10^−5^(*L*)^3.053^	*W* = 2.13⋅10^−5^(*L*)^3.037^	*W* = 6.51⋅10^−5^(*L*)^2.8499^	*W* = 2.02⋅10^−5^(*L*)^2.970^	*W* = 3.45⋅10^−5^(*L*)^2.868^
Growth	*L*_*t*_ = 103.18 (1-*e*^−0.01196(*t*+146.14)^)	*L*_*t*_ = 127 (1-*e*^−0.00228(*t*+63.9)^)	*L*_*t*_ = 129.86 (1-*e*^−0.00439(*t*+96.52)^)	*L*_*t*_ = 142.62 (1-*e*^−0.00717(*t*−60.31)^)	*L*_*t*_ = 167.646*e*^*e*−0.0322504(*t*−87.2819)^	*L*_*t*_ = 303 (1-*e*^−0.00207(*t*+49.4)^)
♀L_50_	73	54	79	84	136	145
L-BF	BF =5.494⋅10^−12^(*L*)^7.6343^	BF =0.0118(*L*)^2.596^	BF =1.5889⋅10^−29^(*L*)^16.1377^	BF =1.2766⋅10^−5^(*L*)^4.1663^	BF =1.8865(*L*)^1.7271^	BF =0.0018(*L*)^3.092^
Size-specific sex ratios	%♀ =232.96 − 1.88(*L*)	%♀ =405.5 − 4.44(*L*)	%♀ =1239.1 − 10.9(*L*)	%♀ =5.99 + 85.49^(−.5∗((*L*−95.58)∕26.92)^∧^2)^	%♀ =346.76 − 1.78(*L*)	%♀ =141.3 − 0.617(*L*)

Isotopic analysis of benthic reef fishes from different feeding guilds in both shallow and MCE habitats of the MHI revealed that carbon isotopic (*δ*^13^C) values for all feeding guilds overlapped across shallow and MCE depth ranges, but some significant differences were observed (Bradley et al., in press). Ranges of *δ*^13^C values in planktivorous fish were smaller than those of benthic invertivores in both shallow and MCE communities. Omnivores showed a greater range in carbon isotopic composition at shallow depths than at MCE depths, and pooled data across O‘ahu and Maui revealed significantly lower *δ*^13^C values for individuals from MCEs compared to shallow individuals (Bradley et al., in press). In addition, significant differences in *δ*^13^C values were found between depths for benthic invertivores, but not in planktivores. Shallow omnivore *δ*^15^N values were slightly (but significantly) higher than MCE omnivores, but no significant differences for the overall populations of invertivores and planktivores were found between depths (Bradley et al., in press). Nitrogen isotopic (*δ*^15^N) values for the majority of source and trophic amino acids (Popp et al., 2007) were not significantly different between depths for any taxon with the exception of *Centropyge* (Pomacanthidae) and *Sargocentron* (Holocentridae), where *δ*^15^N values were lower in MCE fishes compared to shallow fish (Bradley et al., in press). No significant differences in trophic position calculated from amino acid isotopic compositions were found with increasing fish standard length between islands in any feeding guilds. Between depths, amino acid–based trophic positions of MCE benthic invertivores were slightly but significantly higher than those from shallow depths (Bradley et al., in press). For omnivores and planktivores, these results indicate that changes in nutrient sources over the depth range studied did not affect their position within the food web. The small but significantly high trophic position of benthic invertivore feeding guilds from MCEs most likely resulted from consumption of fewer macroalgal grazers on MCEs compared to shallow reefs (Bradley, 2013; Bradley et al., in press).

**Table 5:** Estimates of biomass and egg production for exploited fishes at mesophotic and euphotic depths. MCE column represents the ‘Au‘au Channel, shallow column from previous studies throughout the main Hawaiian Islands (Longenecker & Langston, 2008; Langston, Longenecker & Claisse, 2009).

		MCE	Shallow
*C. potteri*	g/m^2^/yr	0.0773	1.9861
	eggs/spawning event/m^2^	7	26
*C. strigosus*	g/m^2^/yr	0.0382	6.9699
	eggs/spawning event/m^2^	2	340
*P. multifasciatus*	g/m^2^/yr	1.9702	19.9779
	eggs/spawning event/m^2^	133	155

Isotopic results show that individual fish species generally do not differ greatly in trophic position between the two reef ecosystems (Bradley, 2013; Bradley et al., in press), indicating that managing reef fish species as one group across depths may be appropriate. An exception to this general observation was found in a study of diet and movements of Galapagos sharks, *Carcharinus galapagensis* (Snodgrass & Heller, 1905), and Giant trevally, *Caranx ignobilis* (Forsskål, 1775), from a MCE at Pearl and Hermes Atoll in the NWHI. Based on stable isotopic analysis and acoustic telemetry to study diet and movements, Papastamatiou et al. (2015) found that giant trevally occupied a wide range of trophic positions potentially due to intraspecific competition. However, carbon isotopic compositions of several species of benthic feeding fish indicate that carbon flow in the two ecosystems may be distinct (Papastamatiou et al., 2015; Bradley et al., in press). While this does not alter the relative trophic position of the fish, it implies that caution should be taken when considering shallow reefs and MCEs as a single ecosystem as the flow of biomass may be different in the two ecosystems.

### MCEs as refugia

Much has been written about the potential for MCEs to serve as refugia for shallow-reef species (Hughes & Tanner, 2000; Riegl & Piller, 2003; Bongaerts et al., 2010; Hinderstein et al., 2010; Bridge & Guinotte, 2013; Kahng, Copus & Wagner, 2014; Holstein et al., 2015). Most of the discussion has focused on MCEs having reduced susceptibility to coral bleaching events due to reduced irradiance and increased thermal stability, as well as protection from storms and mechanical disturbances (such as anchor damage). To some extent, especially in the MHI where the horizontal distance of many MCEs from shore is large, distance may confer some protection from coastal impacts, such as sedimentation and pollution. In some cases, MCEs may offer protection from fishing pressure, particularly for fisheries that rely on divers or are otherwise impractical at greater depths (Lindfield et al., 2016).

As summarized by Bridge & Guinotte (2013), in order to consider MCEs as refugia for inhabitants of shallow reefs, MCEs must harbor populations of species that are impacted on shallow reefs, in ways that would allow propagules from MCE populations to colonize shallow reef habitat (i.e., adequate genetic connectivity; although potential for propagule dispersion and settlement is not the only determinant of genetic connectivity). MCEs must also be more resilient to stresses that affect shallow reefs. Bongaerts et al. (2010) reviewed the literature regarding the ‘deep reef refugia’ hypothesis for Caribbean reefs and concluded that it is more likely to apply to “depth generalist” species and may serve a greater importance in the upper range of MCEs (30–60 m). This was exemplified by the coral *Seriatopora hystrix* in Okinawa, which was extirpated from shallow water, and later discovered at 35–47 m (Sinniger, Morita & Harii, 2013). A primary goal of our research was to understand the extent of connectivity by species across the archipelago, and both genetic and trophic relationships between MCEs and nearby shallow reef habitats. In the first genetic comparison of shallow and MCE reef fishes, the damselfish *Chromis verator* showed no population structure across depths (Tenggardjaja, Bowen & Bernardi, 2014). Thus, the initial genetic data and the high degree of shared fish species between shallow reefs and MCEs (84%, when considering the full depth range of MCEs) indicate that MCEs may function as refugia for some impacted populations on shallow reefs, especially for fishes (Lindfield et al., 2016). However, biomass and egg production estimates for three exploited species (*C. potteri*, *C. strigosus*, and *P. multifasciatus*) from this study (Table 5) are consistently lower for MCE populations, even though estimates for shallow populations incorporate the effects of fishing mortality. Moreover, patterns of larval dispersal between and among shallow and MCE populations are not well known. Rather than being viewed as a source for shallow-water reef fish, the MCE populations may require more protection than their shallow-water counterparts.

Vertical distribution of scleractinian coral species in the Hawaiian Islands is well known for common, conspicuous species while rare, cryptic or hard to identify species are less understood. Rooney et al. (2010) and Luck et al. (2013) both reported depth ranges for scleractinian species. Based on the anecdotal observations of this study, we observed a similar pattern of species distribution where there appears to be greater species overlap between shallow reefs and upper MCEs (30–60 m) than between shallow reefs and lower MCEs (>60 m). As such, the lower MCE populations do not serve as effective scleractinian species refugia for shallow reefs. This pattern of coral segregation by depth has also been reported by Kahng, Copus & Wagner (2014). Corals in the upper MCE may in some cases serve as refugia for shallower populations, as modeling studies indicate high larval-mediated connectivity (Thomas et al., 2015; Holstein, Smith & Paris, 2016). Few studies have tested genetic connectivity across depths for corals. Based on a microsatellite survey of *Porites astreoides* in the West Atlantic, Serrano et al. (2016) showed high connectivity between shallow and deep reefs in Bermuda and U.S. Virgin Islands, but some evidence of population structure between shallow and deep reefs in Florida. Van Oppen et al. (2011) showed a restriction of gene flow between shallow and deep colonies of the brooding coral *Seriatopora hystrix*, but also some evidence that larvae from deep reefs may seed shallow reefs. Hence the evidence for coral refugia is equivocal at this time, and studies in Hawaii would be valuable contributions to this debate.

Recently, the Caribbean coral, *Porites astreoides*, was shown to have similar reproductive characteristics across depths. Holstein, Smith & Paris (2016) modeled the vertical connectivity of two Caribbean species, *P. astreoides* (brooder) and *Orbicella faveloata* (broadcaster) and predicted significant contribution from both species with a high local contribution from the brooder (Holstein et al., 2015). However, both of these studies were conducted in shallow reefs and the upper MCE and not in the lower MCE further suggesting less connectivity with the lower MCE. Prasetia, Sinniger & Harii (2016) examined the reproductive characteristics of *Acropora tenella* from the upper MCE and found the reproductive characteristics to be similar to shallow reef acroporids, but they did not examine lower MCE colonies.

While MCEs in Hawai‘i may fulfill the first requirement for refugia (at least for fishes and some corals), the resilience of Hawaiian MCEs as compared to their shallow-reef counterparts remains unknown. Several abiotic factors (e.g., exposure to high light or temperature fluctuations) would intuitively cause more stress for shallow reef systems than for MCEs, but the impact of these stressors on coral resilience is not always straightforward. Corals with regular exposure to high temperatures or elevated PAR may be more tolerant of extreme conditions than corals without such exposure (West & Salm, 2003; Grimsditch & Salm, 2006). Conversely, corals inhabiting more stable and cooler conditions on MCEs may be less resilient to temperature changes than their shallow-reef counterparts. The impacts of climate change on MCEs are not yet understood, and we are in need of additional research and predictive modeling before assumptions can be made about the resilience of MCEs as compared to shallow reefs. While vertical thermal stratification maintains some MCEs at lower temperatures than shallow reefs, MCEs may still be vulnerable to thermal anomalies that drive bleaching on shallow reefs. During a September 2014 mass-bleaching event in the NWHI, water temperatures of 24°C were recorded by divers at 60 m at Lisianski, approximately 4°C higher than typical for that depth. Mesophotic coral communities may potentially be as vulnerable to bleaching events as adjacent shallow reefs. However, our ability to predict the oceanographic conditions that cause thermal conditions at MCE depths is more limited than shallow reefs. This threat has potentially severe implications for the numerous undescribed species of algae collected from the same MCE site at Lisianski; with an increased frequency and severity of warm-water thermal anomalies, we may be at risk of losing some of these species to climate change before we even document their existence.

Corals in MCEs are growing at considerably lower SI, and are potentially near the lower limit of light intensity required for photosynthesis. Water clarity was identified as a key factor in predicting the presence of MCEs, with less well-developed MCEs in areas with less light penetration (Costa et al., 2015). It is conceivable that a small increase in turbidity near the surface (e.g., from coastal activities that either produce excess sedimentation directly or increase nutrient levels causing increased plankton densities) could have greater impacts on MCEs than on their shallow-reef counterparts.

As has been suggested previously (Stokes, Leichter & Genovese, 2010; Lesser & Slattery, 2011), the potential for MCEs to serve as refugia for shallow reefs likely depends on multiple factors and should be evaluated on a case-by-case basis for different taxa and different sources of disturbance. One important question that has not been addressed in the previous literature is the extent to which shallow reefs might serve as refugia for MCEs. Given the uncertainties about the relative resilience and insulation from a wide range of environmental stress factors that impact coral-reef environments between MCEs and shallow coral reefs, it is premature to make any assumptions about which habitat is more vulnerable to disturbance or which might serve as a refuge. Thus, when future studies assess the potential for MCEs to serve as refugia to replenish disturbed shallow reefs, they should also consider the implications of the reverse relationship as well.

### Management implications

Our multi-year collaborative research on MCEs across the Hawaiian Archipelago has provided new insights on basic geophysical characteristics, patterns of biodiversity, and information on genetic and trophic connectivity that can enhance the foundations for management and conservation of MCEs and coral-reef environments in general.

The ‘Au‘au Channel is the most extensive complex of MCE coral and macroalgal communities in Hawai‘i. Growing on the island’s deep slope, its fragile structure and biodiversity is currently isolated from some anthropogenic impacts, but MCEs should be considered in all future coastal zone management plans for the region. Currently, part of the ‘Au‘au Channel is listed as a Habitat Area of Particular Concern by the National Marine Fisheries Service. Given its unique geomorphology and biotic characteristics, state or federal managers should include a fully protected area. The discovery of similar MCEs off Kaua‘i, Ni‘ihau, and in the NWHI confirm that such environments occur elsewhere within the Hawaiian Archipelago and likely the broader Pacific. Although these sites have not been studied to the same extent as the ‘Au‘au Channel, it is clear from divers and remote camera surveys that the corals form fragile complexes and would be easily damaged from bottom contact. There also appears to be a rich diversity of mesophotic macroalgae in the NWHI. The need to fully protect the ‘Au‘au Channel and other MCE hotspots within the Hawaiian Archipelago is underscored by the fact that upper MCEs (30–60 m) are likely to serve a more prominent role as a refuge for corals, while the lower MCEs (>60 m) harbor unique assemblages of species with higher rates of endemism. As noted previously, MCEs may also potentially serve as spatial refugia for some overexploited shallow-reef fishes, but this potential must be evaluated on a species-by-species basis.

Our research reinforces the theme that MCEs require clear water, likely due to increased levels of PAR reaching greater depths. These findings indicate that MCEs are less resilient to certain stresses than their shallow-reef counterparts, particularly surface-water clarity. Assessments of the impact of increased turbidity (e.g., from shore-based run-off or nutrient enrichment and its impact on plankton densities) should not be limited to shallow coral-reef ecosystems directly exposed to impaired water quality; it may well be the case that vast expanses of MCE habitat at the limits of photosynthetic production could also be heavily impacted. Examples of dead, coralline algal-covered plate coral formations were observed during this project and highlighted the reality of threats to MCEs.

Other potential impacts to MCE habitats (e.g., fishing pressure, cable laying, placement of permanent moorings and dredging) should be considered in coastal zone management activities, especially when the full extent of MCEs has not yet been documented. For example, with the increasing push for renewable energy and integrated electric grids between islands (undersea cables, offshore windmills, wave energy structures, or other renewable technologies), areas with shallow to intermediate bathymetry, protection from large swells, and a greater distance from shore make certain MCE areas (like the ‘Au‘au Channel) ideal locations for renewable energy structures. Planning for such activities needs to consider vulnerabilities associated with MCEs.

In addition to thermal stress (discussed above), climate-driven threats to MCEs may come in the form of increased frequency and severity of large storms (Emanuel, 2005). Currently, major storms that form in the Eastern Tropical Pacific usually pass south of Hawai‘i, but increasing sea surface temperatures may allow these storms to move north (Pachauri & Meyer, 2015). High benthic cover by corals (*Leptoseris* spp.) on MCEs in the ‘Au‘au Channel is in part facilitated by shelter from wave energy provided by the islands of Maui, Lana‘i, Kaho‘olawe, and Moloka‘i (Costa et al., 2015). A direct hit by a major storm may cause enough mixing of warm stratified surface waters to cause thermal stress at depth, and even a small increase of benthic sheer stress at depth may damage or destroy fragile MCE corals. Finally, ocean acidification may threaten not only corals, but also crustose coralline algae (Jokiel et al., 2008). Rhodolith beds are common features of NWHI and MHI MCEs (Spalding, 2012), and both crustose coralline algae and rhodoliths serve as attachment substrata for MCE algae and antipatharian corals. While there is little that local resource managers can do to alleviate large-scale climate events, it is nevertheless important to understand the potential impacts climate change can have when establishing conservation priorities.

We still know little about MCEs, even in well studied areas such as Hawai‘i, and we do not yet understand the threats to or importance of MCEs in a changing climate. Research needs to be conducted to better characterize whether MCEs will be more or less vulnerable to warming and acidification, or if they will increase the resilience of coral reefs or individual taxa. A better understanding of the basic biodiversity characteristics (e.g., discovering, documenting and describing new species, improving our understanding of depth ranges and endemism, and levels of genetic and trophic connectivity between populations of conspecifics in shallow-reef and MCE habitats) is critical for making informed management decisions. The functional role of MCEs beyond direct connectivity to broader coral reef management must be integrated in future management plans or actions addressing coral reefs.

While this project established core baselines for community dynamics within several regions of the Hawaiian Archipelago, it is vitally important to investigate MCE communities elsewhere throughout the Pacific to provide essential context for comparisons of patterns and processes. Most areas of the Pacific remain completely unexplored. This lack of understanding has profound implications for US waters within the Pacific with the recent listing of 15 coral species under the Endangered Species Act and an additional three species proposed for listing. Several of these species are known to exist in MCEs (Bare et al., 2010) and others may be present, but not documented. Clearly the documentation of MCEs is essential for prudent management of Hawaiian resources, and comparison to other Pacific habitats will lead to a better understanding of the unique biodiversity within Hawaiian waters.

##  Supplemental Information

10.7717/peerj.2475/supp-1 Supplemental Information 1 Detailed Materials and Methods

10.7717/peerj.2475/supp-2 Supplemental Information 2 Detailed methods with Track Changes showing alterations from original submission

10.7717/peerj.2475/supp-3 Supplemental Information 3 Raw data from this study Tab 1: Algae Depth Data; Tab 2: Fish Depth Data; Tab 3: NWHI Fish Trophic Data; Tab 4: Temperature Depth Datasets; Tab 5: Temperature Depth Data.

## Additional Information and Declaration

### Field Study Permissions

The following information was supplied relating to field study approvals (i.e., approving body and any reference numbers): The State of Hawai‘i Department of Land and Natural Resources developed Special Activity Permits for the University of Hawai‘i and National Marine Fisheries Service for work related to this project that occurred within State of Hawai‘i waters. All sampling procedures and experimental manipulations were reviewed as part of obtaining the field permit. Special Activity Permits do not have reference numbers. Papahānaumokuākea Marine National Monument provided research permits for field work in the Northwestern Hawaiian Islands.

### Data Availability

The following information was supplied regarding data availability: Global Biodiversity Information Facility: http://www.gbif.org/dataset/b929f23d-290f-4e85-8f17-764c55b3b284
