# Supplementary material for: A comprehensive investigation of mesophotic coral ecosystems in the Hawaiian Archipelago
Source: PeerJ. 2016 Oct 4;4:e2475. doi: 10.7717/peerj.2475 (PMC5068450; doi:10.7717/peerj.2475)
Supplement: Supplemental Information 1 [file peerj-04-2475-s001.docx]

## Detailed Materials and Methods

The following represents a detailed description of materials and methods used for this study.

### Study Sites

We examined Mesophotic Coral Ecosystems (MCEs) at multiple sites throughout the Hawaiian Archipelago. The primary study sites within the MHI were concentrated in three regions: the ‘Au‘au Channel off Maui, southeast Kaua‘i off Kipu Kai, and the southern shore of O‘ahu (Figure 2). Numerous additional qualitative observations of MCEs in various other sites around the islands of O‘ahu, Kaua‘i, Maui, and Hawai‘i conducted over many years provide complementary insights into general characteristics of MCEs in the MHI. Surveys of MCEs in the NWHI included visits to Nihoa, Necker (Mokumanamana), French Frigate Shoals, Gardner Pinnacle, Maro Reef, Laysan, Lisianski, Pearl and Hermes Atoll, Midway Atoll, and Kure Atoll (see Figure 1).

### Survey Effort

Data from MCEs in the ‘Au‘au Channel were gathered over the course of 100 field days during 13 separate expeditions, involving 57 submersible and Remotely Operated Vehicle (ROV) dives, 86 Towed Optical Assessment Device (TOAD camera sled) transects, and 131 mixed-gas rebreather dives. Data from MCEs off South O‘ahu were gathered from five submersible dives and 16 mixed-gas rebreather dives over a total of eight days of fieldwork, and surveys off Kaua‘i included ten days of fieldwork with 39 open-circuit and rebreather mixed-gas dives. Data from MCEs in the NWHI were obtained over five separate cruises aboard the NOAA ship *Hi‘ialakai* from 2009-2014, and included a total of 131 field days involving 243 open-circuit mixed-gas dives and 228 closed-circuit rebreather dives. The submersible and ROV dives were conducted using the Hawai‘i Undersea Research Laboratory’s (HURL) *Pisces IV* and *Pisces V* submersibles, and the *RCV-150* ROV, deployed from the University of Hawai‘i’s R/V *Ka‘imikai-o-Kanaloa* off Maui, and by the Launch, Recovery and Transport (Kerby 1991) platform off O‘ahu. An additional 265 TOAD dives were made throughout the Archipelago between 2001 and 2011 from several different research vessels, and qualitative observations from several hundred additional mixed-gas dives throughout the MHI from 1989-2014.

Mixed-gas dives were conducted from small shore-based craft in the MHI, and aboard the NOAA Ship *Hi‘ialakai* in the MHI and NWHI. Most dives conducted in the MHI, and dives conducted in the NWHI during 2013-2014 cruises used electronically-controlled mixed gas rebreathers with trimix (helium, nitrogen, oxygen) diluent gas supplies and oxygen partial pressure (PO_2_) setpoint values ranging from 1.3-1.4 atm during the bottom portion of the dive, and a maximum of 1.6 atm during decompression. Rebreather models included Cis-Lunar MK-5P, AP Valves Inspiration, Poseidon SE7EN, and Innerspace Systems’ Megalodon rebreathers. Dives conducted during 2009-2012 in the NWHI and during 2009-2010 in the MHI used mixed-gas open-circuit technology, with trimix for bottom gas and enriched-air nitrox (EAN) and pure oxygen for decompression. Dive sites in the MHI were determined by a variety of factors, including previously known MCE habitat, examination of bathymetry data, and direct site identification by submersible, ROV and TOAD dives. Dive sites in the NWHI targeted steep vertical drop-offs at depths between 50-85 m. These sites were identified using high- to moderate-resolution (5-20 m grid) bathymetry data from the NOAA Pacific Islands Benthic Habitat Mapping Center and the National Geophysical Data Center, and analyzed using ArcGIS software. Additionally, areas without bathymetry data were mapped using the EM3002 Kongsberg multibeam sonar of the NOAA ship *Hi'ialakai*.

On several occasions in the ‘Au‘au Channel and off South O‘ahu, both submersibles and rebreather divers conducted coordinated field operations (Figure 3). In some cases, the submersible was used to identify prospective dive sites and deploy a surface marker buoy for divers to descend (allowing direct access to vetted dive sites, thereby ensuring effective use of rebreather diver bottom time). In other cases, the submersible transported large or bulky equipment to the dive site, and waited while the rebreather divers conducted complex tasks that would have been difficult or impossible for the submersible manipulator arm to complete. The submersible also located an appropriate site then remained at the site while divers descended to collect fish and invertebrate specimens.

This study did not involve any endangered or protected species. The State of Hawai‘i Department of Land and Natural Resources developed Special Activity Permits for the University of Hawai‘i and National Marine Fisheries Service for work related to this project that occurred within State of Hawai‘i waters. All sampling procedures and experimental manipulations were reviewed as part of obtaining the field permit. All vertebrates (fishes) were collected in accordance with University of Hawai‘i IACUC protocol 09-753-5, “Phylogeography and Evolution of Reef Fishes” (PI: Dr. Brian Bowen), including collection and euthanization by spear.

### Geophysical Habitat Characterization

The majority of geophysical habitat characterization focused on the ‘Au‘au Channel site. Existing moderate-resolution (20-m grid) bathymetry was supplemented with multibeam surveys at a resolution of 5 m in a few areas. Extensive video transects from submersible, ROV and TOAD dives were used to document the spatial distribution of corals and macroalgal communities. Habitat was appraised using a live feed video camera (ROV or TOAD) to survey habitat between ~30 and 150 m, covering more than 140 linear km of habitat (Figure 2). The distributions of these communities were mapped in detail and plotted using GIS tools, and the results were used extensively for planning subsequent dives and other research activities.

Two specific areas (“John”, at a site with high coral cover on the rim of a solution basin, and “Frank”, at a nearby site without coral in the center of the solution basin) were selected for detailed physical oceanographic characterization. The John mooring was deployed on August 5, 2008 at 84 m depth (20° 46.688 N, 156° 40.417 W); the Frank mooring was deployed on August 7, 2008 at 123 m depth (20° 46.297 N, 156° 40.241 W; Figure 2 inset). A total of four oceanographic moorings were deployed (Figure 4). The two primary moorings (John and Frank) were set up identically, with Seabird 39 temperature and pressure loggers (1 m off bottom), an Aanderaa single point current meter (2 m off bottom), an upward-facing Sontek 250 khz current profiler (4 m off bottom), and 3 Onset Computer Corp TidBit temperature recorders (10 m, 20 m and 30 m off bottom). All instruments were set to log internally and recorded good quality datasets for the duration of the deployment. The Aanderaa current meter recorded current speed and direction at a single point in the horizontal plane every 10 minutes. The Sontek recorded a profile of current speed and direction in three dimensional space every 20 minutes; these profiles were recorded in 25 cells, each 2.5 m, with a 1.5 m blanking distance from the instrument head. The Seabird 39 temperature and pressure logger recorded a single point temperature and pressure reading every 10 minutes, and the TidBit temperature recorders captured readings every 20 minutes. The timers on the data recorders were synchronized to start each recording interval simultaneously. The length of the mooring lines, the closeness of the deployments and the depth placements of the moorings allowed consideration of the data as a single long mooring, measuring approximately the same water mass instantaneously. This is supported by the current profile data and temperature data that, at the same depth, have similar current regimes and temperature profiles. The overlap between moorings encompasses approximately the upper 25 m of the “Frank” mooring, and the deeper 25 m of the “John” mooring, providing year-long monitoring of the thermal structure of the water column. Bottom temperature was also collected at the base of two other short 5 m “Tele” moorings. Recordings from all four moorings provided a thermal cross-section of the contour, including shallow and MCE habitats with and without coral.

Additional temperature data were recorded at four other sites across Maui and Kaua‘i (Table 1). The temperatures were recorded with Onset TidBit v2 Water Temperature Data Loggers (UTBI-001) attached to the substrate and set to record at 30-minute intervals, and sensor deployment ranged from 9 to 13 months.

Underwater irradiance was measured by lowering a calibrated spherical (4π) quantum sensor (Underwater LI-193SA, LI-COR, Lincoln, NE, USA) through the water via a profiling rig (n = 6 profiles taken within one hour of high noon on 4, 5, and 6 August 2008 and 12, 13, and 14 July 2010 over dense *Leptoseris* spp. reefs at a maximum depth of 91 m depth off west Maui); data were stored with a LI-COR LI-1400 datalogger. The sensor was attached to a 1 m long arm mounted on a polyvinyl chloride housing to reduce instrument shading. The housing was integrated with a calibrated pressure transducer for depth (m) and a temperature sensor. PAR (μmol photons m^-2^ s^-1^) was recorded every 0.2 to 0.5 m in the water column to a maximum depth of 91 m on calm, clear days. Profiles were done on the sunny side of the vessel to reduce any shadows. The vertical attenuation coefficient (K_o_) from the downward portion of each profile was calculated according to the relationship in Beer’s Law:

I_z_ = I_o_ x e^-kz^

where z represents depth, I_z_ represents the intensity of irradiance at depth z, and I_o_ represents the intensity of irradiance just beneath the surface (Kirk 2011). The percent of subsurface irradiance (% SI) at any one depth was extrapolated from the average K_o_ from the 6 profiles. The average irradiance ± Standard Error (SE) was calculated by averaging irradiances at specific depths from each site based upon the K_o_ determined for each site.

### Biodiversity Patterns

The biodiversity surveys focused primarily on macroalgae, fishes, marine invertebrates, and corals. Surveys were conducted using a variety of visual, video, and collecting techniques. Direct visual observations were made by trained individuals during mixed-gas SCUBA dives and from submersibles. Videotapes were generated by divers, submersibles, the ROV, and the TOAD camera system. Specimens were collected by divers and using the manipulator arm of the *Pisces* submersibles (Figure 5).

Survey sites were chosen to represent several habitat types, including rocky limestone ledges with undercuts and caves, rubble fields, *Halimeda* meadows, other macroalgal assemblages, coral and sand fields, branching coral reefs, *Leptoseris* reefs, and open sand. Comparative shallow-water (<30 m) surveys were conducted at various sites off Maui and O‘ahu in the MHI, and at all of the ten major reefs, islands and atolls in the NWHI.

Qualitative collections and observations were made to determine species presence, and quantitative transects were made to measure the distribution and abundance of species. In the MHI, qualitative transects were made using both the submersibles, and mixed-gas divers, either recording direct visual observations or using hand-held video cameras. The submersible transects were conducted at depths between 42 and 91 m, and varied in distance by following a specific isobath and habitat for up to 20 minutes. With the sub cruising at 2 knots at 1 m above the bottom, all fish encountered within the width of area of the submersible’s viewport (6 m) were identified to the most precise taxon possible, counted, and their body lengths estimated using 5-cm categories. A laser reference scale was projected on the bottom within the view of the video cameras to assist the observers in fish length estimations. Diver surveys counted all fishes within 1m on either side of the transect line (25 m x 2 m belt). Videos from ROV and TOAD transects were used to quantitatively assess the relative abundance of species across larger geographic scales. Substrate type (sand, rubble, rock, etc.) and living cover (macroalgae, hard corals, coralline algae, etc.) were identified from point counts every 30 seconds in the video files (mean distance of ~12 m between points). All quantitative NWHI fish surveys were conducted using a visual diver survey of a 25 x 2 m belt transect, complemented by qualitative presence/absence observations (Kane, Kosaki and Wagner 2014).

Representative macroalgae samples were collected from 59 sites at depths of 40-212 m around the MHI of O‘ahu, Maui, Lāna‘i, Kaho‘olawe and Moloka‘i (Spalding 2012). Although total algal survey effort was not consistent at all depths, care was taken to determine the greatest depth limit of algal occurrence for every dive. We are confident that our results for species composition are representative of the diversity at each of the depths sampled.

Coral rubble collected on submersible dives was kept alive in bubbled water and cultured in the laboratory for epiphytes. Algae were identified morphologically using authoritative works (Abbott 1999; Abbott and Huisman 2003; Huisman, Abbott and Smith 2007), and verified by taxonomic experts (see Spalding 2012). Macroalgae from the NWHI were collected by mixed-gas divers, saved in triplicate on the boat (4% formalin in seawater, silica gel for molecular analyses, and frozen for pressing), and processed for stable isotopic analyses. Frozen algae were defrosted and pressed at the Department of Botany, University of Hawai‘i at Mānoa for taxonomic work.

Corals belonging to the genus *Leptoseris* (n=76) were collected across multiple depth gradients (65–125 m) from 31 different sites in the MHI, between the islands of Maui and Lāna‘i (Pochon et al. 2015). At each site, representative *Leptoseris* corals approximately 20-30 cm in diameter were haphazardly selected from the middle of a *Leptoseris* reef, with each sample separated by at least 10 m in distance. A small, triangular piece of coral spanning from the middle to the outer edge of the coral head was removed using a Schilling Titan 4 manipulator arm on the submersible, and placed in an individual sample container in the sampling basket. Branching *Montipora* samples (n=22) from 3 sites off west Maui were collected from 56 to 67 m depths by mixed-gas divers and the submersible, respectively. Collected samples were kept in a darkened container with ambient seawater and *in situ* temperatures, and processed in a darkened laboratory within 4 h of ascent to the surface. Each sample was photographed, sampled for coral and bacterial DNA, and then immediately frozen at −80°C. Coral and *Symbiodinium* genotypes were compared with three distinct molecular markers including coral (COX1–1-rRNA intron) and *Symbiodinium* (COI) mitochondrial markers and nuclear ITS2 (Pochon et al. 2015).

Invertebrate specimens were extracted from collected rocks and corals, after being brought to the surface. Other cryptic invertebrates were incidentally collected with live coral, rubble, and other invertebrate and algae samples. Two sand/rubble scoops were taken near live coral areas and closed off at depth to collect infaunal and cryptic invertebrates. Gammaridean amphipods were removed from all biological samples collected during submersible operations.

Fish specimens were only collected in cases where vouchers were necessary for taxonomic identification, for documenting important depth or geographic records, or to obtain samples for genetic and isotopic analyses. Fishes were collected from various sites throughout the MHI and NWHI, primarily with the use of hand nets and in some cases rotenone.

All specimens obtained from field surveys were photo-documented using high-resolution digital cameras, and voucher specimens were deposited in the Bishop Museum Natural Sciences collections. Samples were analyzed primarily at the University of Hawai‘i (Department of Botany, Department of Geology and Geophysics, and the Hawai‘i Institute of Marine Biology) and the NOAA Pacific Islands Fisheries Science Center.

To analyze faunal and floral breaks across different depths, we examined patterns of species changeover for both fish and macroalgae at 10-m depth intervals down to 150 m. Our surveys during this study were not complete enough or comprehensive enough to analyze beta-diversity using traditional approaches (Koleff, Gaston and Lennon 2003), whereby presence/absence within ecological divisions such as depth zones are quantitatively analyzed and compared. Instead, we took a more holistic approach to known species depth ranges of species across the Hawaiian Islands, and examined minimum and maximum depths for each species. Species depth ranges were determined as the maximum and minimum depth values reported in the literature (Abbott 1999; Mundy 2005; Randall 2007), within specimen and occurrence databases (e.g., Bishop Museum collections data, Explorer’s Log database [http://explorers-log.com]), and data gathered from surveys, images, video, and observations obtained during this project. At each depth interval, the number of species with a reported maximum depth within 10 m above the interval depth were added to the number of species with a reported minimum depth within 10 m below the interval depth (representing the total number of species participating in a changeover at that depth interval). This summed value was divided by the total number of species that occur at the depth interval, to yield a percentage of species that participate in a changeover. Larger values indicate a more substantial break; smaller values indicate a less substantial break. Percentage of total species within a depth zone was used instead of absolute numbers of species because we were specifically interested in comparing the proportion of species change at different depths as a measure of relative change. Using absolute species numbers would artificially bias the amount of change due to differences in alpha diversity.

### Population Dynamics

A *Leptoseris* coral colony at a depth of 83 m was covered with a special Plexiglas dome equipped with syringes of seawater and Alizarin Red stain, deployed by the *Pisces* submersible on December 9th, 2007. The alizarin red stain solution was injected into the dome to produce a concentration of 20 ppm, and an electric CTD pump was used to circulate water within the dome. The submersible returned approximately 6 hours later to remove the dome, by which time most of the stain had come out of solution and settled (Figure 6). This colony was recovered two years later (January 19th, 2010) for examination of banding and X-radiographic imaging. Accumulated bands were then compared with the cycle of temperature over the two years logged by a thermograph (Onset TidBit v2) placed next to the marked coral. Additional colonies of *Leptoseris* were stained *in situ* at a depth of 88 m using the same Alizarin Red staining technique involving the Plexiglas dome, facilitated by coordinated rebreather diver and submersible dives. Temperature loggers were deployed next to the corals when they were stained. These additional stained colonies were left in-situ to assess growth patterns over longer durations, and will be recovered as part of a future study. Additional test colonies of *Leptoseris* collected in nearby were sent to collaborators at the Woods Hole Oceanographic Institution for computerized axial tomography (CT) scanning, ^14^C and U/Th (Uranium-thorium) dating and elemental ratio analyses to determine growth rates.

Three fish species exploited on shallow reefs and reported from MCEs, *Centropyge potteri*, *Ctenochaetus strigosus*, and *Parupeneus multifasciatus*, were selected to compare estimates of production and reproductive output on shallow-reef versus MCE habitats. Data for populations on shallow reefs were generated during previous studies (Longenecker and Langston 2008; Langston, Longenecker and Claisse 2009). For populations on MCEs, 37 *C. potteri*, 33 *C. strigosus*, and 33 *P. multifasciatus* were collected from MCE depths in the ‘Au‘au Channel. These specimens were used to generate length-weight and length-fecundity relationships, and to estimate size-at-maturity using the methods of Longenecker et al. (2013). These specimens were also used to describe size-specific sex ratios using the method of Longenecker et al. (2014). A single, transverse section of each sagitta was prepared and examined to describe growth (Longenecker and Langston 2006). Otolith preparations rarely included the primordium, so age was estimated by counting the number of increments past an easily identifiable check mark for each species, and adding an assumed number of days for the region inside the mark. The check mark corresponded to 36 d for *C. potteri* (Thresher and Brothers 1985), 76 d for *C. strigosus* (Langston, Longenecker and Claisse 2009), and 21 days for *P. multifasciatus* (Longenecker and Langston 2008). Growth II (Henderson, Seaby and Somes 2006) was used to construct vonBertalanffy growth curves for *C. potteri* and *C. strigosus*, and a Gompertz growth curve for *P. multifasciatus*. We also conducted 21 laser videogrammetry surveys (Figure 7) to estimate densities and describe the size structure of the target species in the ‘Au‘au Channel. For each survey, a pair of divers swam a constant heading for a distance limited by bottom time and influenced by current speed and direction. Start and stop points of each transect were estimated using a GPS receiver towed on a surface float. To estimate fish densities, one diver counted all target species individuals observed within a 4-m wide swath along the transect, for a total of 16,278 m^2^ surveyed. To describe size structure, the second diver used a laser videogrammetry apparatus to record all individuals encountered while maintaining sight contact with the dive partner (i.e., laser videogrammetry was not constrained to the 4-m belt transect).

Growth equations were used to convert lengths (estimated from laser videogrammetry) for 139 individuals (21 *C*. *potteri*, 28 *C*. *strigosus*, and 90 *P*. *multifasciatus*) to age estimates. Separate growth equations were constructed for populations of each species on shallow reefs and MCEs (i.e., growth rates were not assumed to be the same for fishes in both depth ranges). Mortality was then estimated from the resulting age distribution (Everhart and Youngs 1992). We assumed MCE populations were not fished (although some fisheries do exploit MCEs, none target these species), therefore total mortality was equivalent to natural mortality (M). Growth equations, mortality estimates, and length-weight relationships were used in a Ricker production model (Everhart and Youngs 1992) to estimate annual biomass production, with the assumption that mortality was constant over all age classes. Observed abundance and age-structure was used to choose the number of recruits in the starting point of the model; it was assumed that the subset of individuals counted during belt transect censuses had the same age structure observed during laser-videogrammetry surveys. The percentage of individuals in the most populous age group was multiplied by the total number of individuals encountered during belt transects to determine the expected number of individuals in the most populous age class. The number of recruits needed for the Ricker model was iteratively determined to produce the same number of individuals in the same age class. The Ricker model was modified to estimate egg production per reproductive event. The number of individuals in each age class was estimated by dividing cumulative weight by average individual weight. Growth equations were used to estimate average individual length in each age class, with the assumption that egg production was zero in all groups containing individuals shorter than female *L_50_*. In age groups containing individuals larger than female *L_50_*, we used size-specific sex ratios to estimate the percentage of females and ultimately the number of reproductive females. Egg production per reproductive event was estimated by multiplying the results of length-fecundity equation by the expected number of reproductive females. Biomass and egg production estimates were standardized by dividing by total area surveyed to permit comparisons between shallow and MCE populations.

### Broad Trophic Characterizations

To determine the trophic level of key food web components and functional groups, we conducted stomach content and carbon and nitrogen stable isotopic analyses on more than 750 reef fishes from 45 species, 30 genera and 18 families collected from both shallow reefs and MCEs in the MHI off the coasts of Maui and O‘ahu from 2008 to 2011. Species were grouped into feeding guilds consisting of omnivores, benthic invertivores, planktivores, and piscivores based on existing stomach content analysis (Bradley et al. 2016). Of the collected specimens, 24 species from seven families were selected for further comparison due to their abundance in both shallow-reef and MCE communities, resulting in 366 samples for bulk tissue isotope analysis; 84 of these samples were chosen for more detailed nitrogen isotopic analysis of individual amino acids (Bradley et al. 2016). We used compound-specific isotope analysis of amino acids because it is a technique that avoids many of the shortcomings of traditional bulk tissue isotope analyses to estimate trophic positions of fishes. (McClelland and Montoya 2002; McClelland, Holl and Montoya 2003; Pakhomov et al. 2004; Popp et al. 2007; McCarthy et al. 2007; Hannides et al. 2009).We used the difference between the nitrogen isotopic compositions of trophic and source amino acids to calculate trophic position Chikaraishi et al. 2009; Bradley et al. 2016).

In addition, 12 Galapagos sharks [*Carcharhinus galapagensis* (Snodgrass & Heller 1905)] and 25 giant trevally [*Caranx ignobilis* (Forsskål 1775)] were collected for bulk tissue (all specimens) and compound-specific amino acid isotope analysis (all sharks and 11 trevally) using baited hand lines at Pearl and Hermes (NWHI) during two research cruises in the summer of 2011 and 2012. For a proportion of the shark and trevally, after a ~1 g sample of white muscle was taken for isotope analyses, we implanted an acoustic transmitter into the body cavity along the ventral surface of the fish through a 2-3 cm incision and closed the incision using a single suture (Papastamatiou et al. 2015). Forty eight potential prey consisting of four species of reef fish were also collected for bulk tissue isotope analysis from Pearl and Hermes. Bulk tissue isotope analysis was performed using an on-line carbon-nitrogen analyzer coupled with an isotope ratio mass spectrometer (Popp et al. 2007).

Samples were prepared for amino acid compound-specific isotopic analysis and analyzed using techniques described by Hannides et al. (2009) andHannides et al. (2013). Species of fishes in the NWHI surveys were grouped into seven feeding guilds consisting of planktivores, sessile invertivores, piscivores, omnivores, mobile invertivores, herbivores, corallivores, and apex predators (Papastamatiou et al. 2015), to allow comparison of relative representation of trophic guilds on both shallow reefs and MCEs.

## References

See References section of main article.
